# High-throughput analyses of a reconstituted diversity-generating retroelement identify intrinsic and extrinsic determinants of diversification

**DOI:** 10.1371/journal.pgen.1012038

**Published:** 2026-02-05

**Authors:** Irem Unlu, Marina K. Smiley, Vladimir Potapov, Yoan Renoux-Martin, Zhi-Yi Sun, Hoong Chuin Lim

**Affiliations:** 1 Research Department - RNA, New England Biolabs, Ipswich, Massachusetts, United States of America; 2 École Supérieure de Biotechnologie de Strasbourg, Strasbourg, France; University of Lausanne: Universite de Lausanne, SWITZERLAND

## Abstract

Diversity-Generating Retroelements (DGRs) are specialized genetic systems typically harnessed in nature to evolve new molecular recognition. This mechanism, known as mutagenic retrohoming, relies on an error-prone reverse transcriptase (bRT) that introduces errors at template adenines, followed by the incorporation of the resulting mutagenized complementary DNA (cDNA) into a homologous target gene. Although widely distributed, DGRs are conspicuously absent from key bacterial models, limiting our understanding of their functionality in these hosts and their potential as engineering tools. Here, we demonstrate the ‘plug-and-play’ nature of the *Bordetella* phage BPP-1 DGR by successfully reconstituting the mutagenic retrohoming mechanism in *Escherichia coli*. Using high-throughput tools available in this tractable bacterium, we identified key regulatory factors that allowed us to enhance DGR efficiency over 1000-fold. Systematic analysis defines how sequence context governs bRT’s fidelity, uncovering a distinct error profile for the AAC motifs prevalent in natural DGR templates. This intrinsic bias prioritizes the sampling of residues essential for antigen recognition, effectively focusing the evolutionary search within the most productive regions of sequence space. Furthermore, a transposon sequencing screen identified the single-stranded DNA exonuclease ExoI as an inhibitor of DGR activity. While removing ExoI enhanced activity by more than ten-fold, we found that its nuclease activity was dispensable for this inhibition, suggesting a non-catalytic mechanism. Finally, a genome-scale survey highlighted enhanced DGR efficiency at targets located near the replication origin and oriented outwardly from it. This bias is clearly linked to replication directionality, suggesting that certain aspects of DNA replication cycles promote mutagenic retrohoming. Collectively, our work reveals previously unappreciated mechanistic features of DGRs and establishes this reconstituted system as a powerful platform for targeted gene diversification and clarifying the molecular mechanism of mutagenic retrohoming.

## Author summary

Our study focuses on Diversity-Generating Retroelements (DGRs), a biological “evolution engine” that microbes and viruses use to rapidly develop new functions within specific genes. DGRs work by using a sloppy enzyme that intentionally makes mistakes as it converts an RNA sequence into a new DNA strand. This mutated DNA is then incorporated into a target gene, creating a library of diverse variants from which new, beneficial functions can arise. To study this more effectively, we moved a viral DGR system into a common laboratory bacterium *Escherichia coli*. Using high-throughput methods, we discovered how the system tilts the scales towards mutations that help proteins recognize new targets. We also found ways to boost this activity by 1000-fold. Finally, we found that DNA replication is a major driver of how efficiently the DGR works, offering new clues into how the mutations are incorporated into the target gene. By uncovering these rules, we provide a new roadmap for fine-tuning this “engine” for future applications in biotechnology.

## Introduction

Life constantly innovates through genetic trial and error. Organisms explore the vast genetic landscape by accumulating mutations to discover novel traits and adapt to changing environments. This exploration can be sped up through increased mutation rates, but these risks compromise genome integrity. Therefore, strategies enabling organisms to mutate rapidly but safely confer a profound evolutionary advantage, especially when a precise adaptation is urgently needed.

This delicate balance is exemplified by how our immune system generates antibodies to target foreign invaders. A vast array of unique antibodies is created by restricting heightened levels of DNA rearrangement and mutations to the antibody genes, sparing the rest of the cell’s DNA. Another distinct strategy for rapid, targeted adaptation involves diversity-generating retroelements (DGRs), found in phages, bacteria, and archaea [1–4]. For example, the *Bordetella* phage BPP-1 employs DGR to selectively mutagenize adenines within the *mtd* (major tropism determinant) gene, which encodes the tail fiber protein responsible for host recognition [1]. This generates a large repertoire of Mtd variants, promoting adaptation to the changing cell surface of its bacterial host [1].

**Fig 1:**
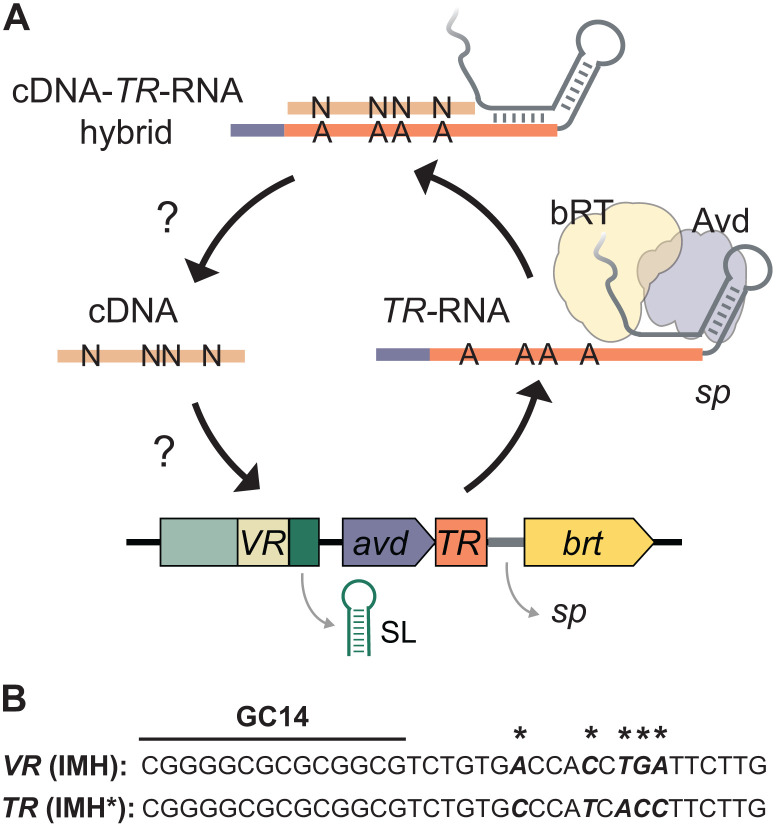
Mechanistic model of mutagenic retrohoming. **(A)** The BPP-1 DGR mediates mutagenic retrohoming using four core components: a reverse transcriptase (bRT), an accessory variable determinant (Avd), and two highly similar sequence repeats, namely the template repeat (*TR*) and variable repeat (*VR*) [2]. The *TR*-RNA, which serves as the template, is flanked at the 5’ end by the coding sequence of *avd* and its 3’ by a spacer sequence (*sp*) located between *TR* and *brt*. This spacer sequence folds into an intricate structure that recruits the bRT-Avd complex [8]. An internal adenine within this spacer then primes the initiation of cDNA synthesis within the 3’ junction of *TR* known as IMH* [6], resulting in a covalently attached cDNA-*TR*-RNA hybrid. A sequence motif within the 3’ end of the adjoining *avd* mRNA terminates reverse transcription [9]. Due to the unique error-prone nature of bRT, the resulting cDNA contains mutations opposite template adenines [6,9]. The precise mechanism by which this mutagenized cDNA then ‘retrohomes’ into the *VR* to modify the target gene remains incompletely understood. However, this process is known to be facilitated by two additional elements: 1) IMH located at the 3’ end of *VR*, which is nearly identical in sequence to IMH* (corresponding sequence in the BPP-1 DGR are shown in **(B)** [1]; 2) An inverted repeat that can form a stem-loop structure (SL) is located immediately 3’ of IMH [10].

DGRs diversify target genes through a process called mutagenic retrohoming [2]. Extensive studies on the BPP-1 DGR have defined the operating principles of this mechanism (Fig 1). Researchers have identified and characterized the dedicated reverse transcriptase (bRT) and its error-prone activity as the source of sequence diversity [5–9]. They have also revealed the sequence elements crucial for target recognition and discrimination [5,6,10] and clarified how template RNA controls the initiation and termination of complementary DNA (cDNA) synthesis [8,9].

Despite significant progress, several aspects of this gene diversification system remain unclear. We do not fully understand the mutagenic propensity of the bRT, particularly how its fidelity is influenced by the surrounding template sequence context. Once the mutagenized cDNA is synthesized, the exact process by which it is integrated into the target gene remains unknown, including whether this process requires additional, currently unidentified, host-specific factors. Furthermore, it is uncertain why this powerful gene diversifier is absent in many bacteria, including model bacteria like *Escherichia coli* and *Bacillus subtilis* [11–13]. It is unclear whether this omission is an evolutionary accident or if it reflects an underlying incompatibility between these host environments and DGR function.

Here, we successfully reconstituted the *BPP-1* DGR system in *E. coli*, demonstrating its functional transferability to an evolutionarily distant host. Using high-throughput analyses, we have uncovered an intrinsic bias in bRT that creates an evolutionary “shortcut” for the rapid generation of functional receptors. Furthermore, our identification of an endogenous inhibitor and the discovery of target location and orientation effects offer ways to manipulate DGR efficiency and new insights into the mechanism governing cDNA integration. By defining these intrinsic and extrinsic determinants, our work provides a new framework for a deeper understanding of mutagenic retrohoming and the design of programmable diversification systems.

## Results

### Reconstitution of the BPP-1 DGR in *E. coli*

**Fig 2:**
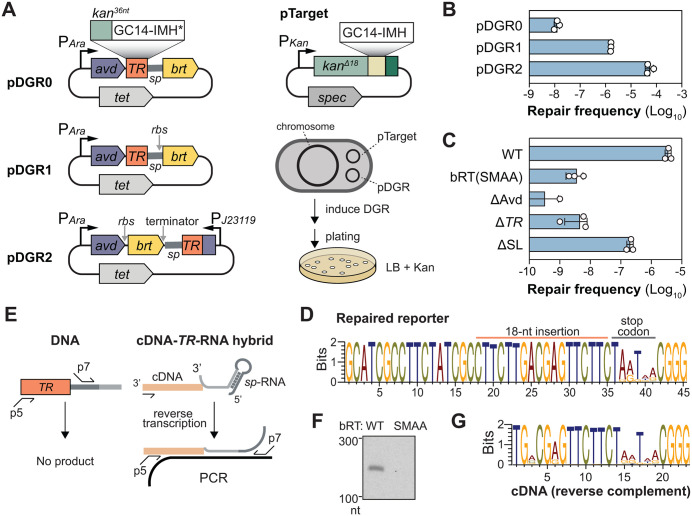
Reconstitution of BPP-1 DGR-mediated editing in *E. coli.* **(A)** Schematic of the DGR-mediated kanamycin repair reporter assay [63]. Left, Different pDGR variants, which encode all essential DGR components except for the target. The *TR* consists of the last thirty-six nucleotides of the *kanR* gene, followed by tandem stop codons (TAATAA). Right, pTarget encodes the target gene, which is a defective *kanR* gene terminated by TGATAA. DGR-mediated repair of the reporter was monitored by quantifying the frequency of kanamycin-resistant clones (repair frequency)*.* **(B)** Bar graph showing the repair frequency for pDGR0, pDGR1, and pDGR2 in *E. coli*. Each of these plasmids was used in tandem with pTarget to transform *E. coli* strain MG1655. Freshly transformed colonies were used to inoculate LB + Tet10 + Spec50. The cultures were grown for 1.5 hours at 37°C, after which arabinose was added to induce expression. The cultures were then grown for an additional 4.5 hours. Finally, the cells were then plated on rich media agar supplemented with Kan25 to select for resistant clones. See Methods for details. **(C)** Bar graph comparing the repair frequency of pDGR1 and variants with the indicated mutations and deletions. These include: bRT(SMAA), YMDD>SMAA active site mutations in *brt*; Δa*vd,* three nonsense mutations in *avd*; Δ*TR*, *TR* deletion; ΔSL, stem-loop deletion. Data are presented as mean ± standard deviation (n = 3 biological replicates). **(D)** Weblogo showing the recovery of the missing 18-nt in pTarget upon induction of DGR expression. Plasmids were harvested from a pool of kanamycin resistant clones containing both pTarget and pDGR1. The plasmids were then used as a template, and the target region was amplified by PCR for deep-sequencing. Only sequencing reads showing an 18-nucleotide insertion in the reporter were used to generate the Weblogo. **(E)** Scheme for specifically amplifying the cDNA-*TR*-RNA for deep-sequencing while avoiding DNA template amplification. **(F)** Agarose gel showing the RT-PCR product is dependent on an intact bRT. **(G)** Weblogo showing the results of cDNA sequencing. The reverse complement of the cDNA sequence is shown to facilitate easy comparison to the reporter in panel **D**. This sequence is shorter because the primer binding region is excluded.

We set out to reconstitute the archetypal BPP-1 DGR in *E. coli*, leveraging the extensive genetic tools available in this bacterium to better understand the mechanism of DGR. We encoded the essential BPP-1 DGR components on two plasmids (Fig 2A): pDGR0 carried the *avd-TR-brt* operon under an arabinose inducible promoter, while pTarget contained a target gene immediately followed by the essential regulatory elements: GC14, IMH, and stem-loop.

To assess DGR activity, we used a reporter system previously used to characterize the BPP-1 DGR in its native host [10]. In this assay, the target gene was a defective kanamycin resistance cassette lacking the sequence for its last six amino acids. The *TR* was programmed with the sequence encoding the final twelve amino acids of the full-length cassette (Fig 2A). This design should enable DGR to transfer the missing sequence to the target through a cDNA intermediate, thereby “repairing” the defective kanamycin resistance gene. We then measured DGR activity by quantifying the frequency of kanamycin-resistant clones, which we termed the repair frequency.

Our initial attempts to reconstitute DGR activity in *E. coli* MG1655 using pTarget and pDGR0 yielded kanamycin-resistant clones at a meager ~10^-8^ frequency (Fig 2A–2B). Absence of DGR function likely stemmed from the lack of a clear ribosomal binding site (RBS) upstream of the *brt* gene in the native BPP DGR operon. Introducing a consensus *E. coli* RBS before *brt* in the new construct (pDGR1) increased kanamycin-resistant colonies 100-fold (Fig 2B). Reasoning that *TR*-RNA level might limit DGR activity, we created pDGR2 (Fig 2A). In this construct, we removed the *TR-*RNA from the operon and placed it under the control of P_J23119_, a strong constitutive synthetic promoter often used for expressing single-guide RNAs in CRISPR applications [14]. This modification improved repair frequency by 35-fold over pDGR1 (Fig 2B), a result likely mediated by the elevated production of both TR-RNA and bRT in pDGR2 (S1 Fig).

This reconstituted DGR system recapitulates key genetic features observed in the native host. The gain of kanamycin resistance did not occur through homologous recombination (S2 Fig), but requires intact essential DGR components: inactivating bRT, Avd, or *TR* reduced repair frequency to ~10^-9^ (Fig 2C). For reasons that remain unclear, repair frequency was reproducibly lowest for DGR lacking *avd*. Additionally, deleting the inverted repeat downstream of IMH severely—but not completely—impaired repair. In *Bordetella*, residual DGR activity was also observed in the absence of this inverted repeat. These results confirmed that this stem-loop motif is not strictly required for mutagenic retrohoming but is clearly important for optimal efficiency (Fig 2C).

To directly confirm that resistance was acquired through DGR-dependent repair of reporter, we deep-sequenced the reporter isolated from resistant cells. Roughly half of the *VR*s have recovered the missing 18 nucleotides (S3A Fig). This heterogeneity reflects the co-existence of repaired and unrepaired pTarget within the same cells, as Sanger sequencing of pTarget from individual clones often showed sequence heterogeneity at the site of insertion (S3B Fig). In contrast, only 3–4% of pTarget isolated from background kanamycin-resistant clones (from our catalytically inactive bRT mutant control, arising at a frequency of <10^-8^) acquired the missing sequence (S3A Fig). Thus, *TR*-*VR* recombination is rare, and the majority of background resistance likely emerged through suppressor mutations elsewhere in the genome. Collectively, these results confirm DGR-dependent restoration of the reporter.

The sequencing analysis also identified the characteristic A-to-N mutations within the restored reporter (Fig 2D). Mutations were limited to the four adenines in the stop codons, even though cDNA sequencing showed that bRT produces significant mutations throughout the cDNA (Fig 2E–2G). Because programmed synonymous mutations at non-adenine positions were incorporated at 100% efficiency without affecting the repair frequency (S4A-B Fig), we conclude that our selection excluded missense mutations in coding adenines due to reporter inactivation. Consequently, the observed repair frequency underestimated the true frequency of DGR-mediated target editing.

Together, these results demonstrate that DGR’s characteristic mutagenic retrohoming activity can be faithfully reconstituted in *E. coli* with its core components alone.

### Sequence context biases nucleotide incorporation for A-to-N mutations

**Fig 3:**
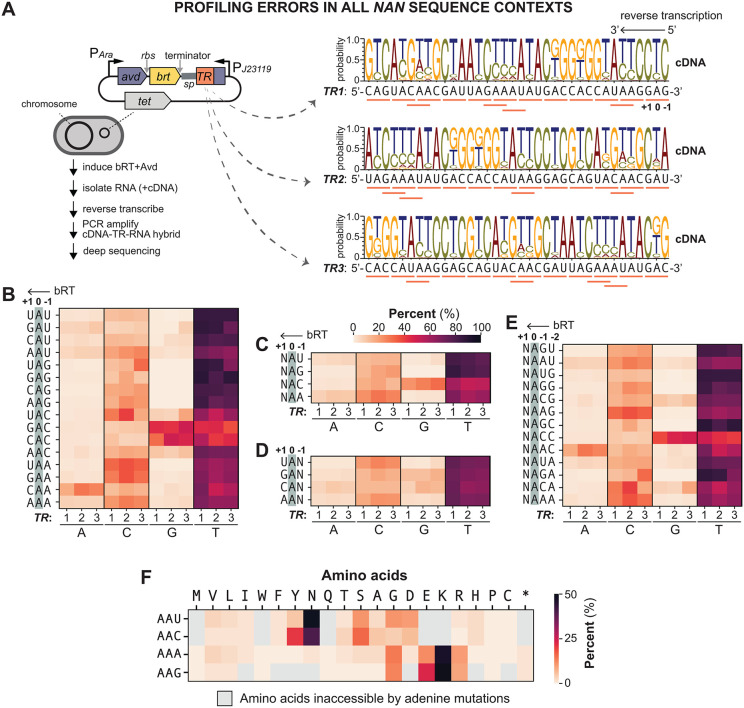
Effect of sequence context on the fidelity of BPP-1 bRT. **(A)** Left, Scheme for cellular profiling the mutational signature of bRT across various template sequence contexts. Right, pDGR2 was programmed with *TR1*, *TR2*, and *TR3*, each containing all 16 possible NAN triplets (underlined in orange) in different orders. Weblogos display the error profiles of cDNA synthesized by bRT from *TR1*, *TR2* and *TR3* RNA templates. Each WebLogo was generated from 10 million subsampled reads pooled from triplicate sequencing runs. **(B)** Heatmap shows the percentage of A, C, G and T incorporation opposite template adenines within all 16 NAN contexts, using *TR1*, *TR2* and *TR3* as RNA templates. The Y-axis shows the RNA sequence contexts. **(C)** Heatmap shows the percentage of base incorporations opposite template adenines in the NAU, NAG, NAC and NAA contexts, highlighting the effect of the nucleotide immediately 3’ (-1 position) of the adenine. **(D)** Heatmap shows the percentage of base incorporations at template adenines in the UAN, GAN, CAN and AAN contexts, highlighting the influence of the nucleotide immediately 5’ (+1 position) of the adenine. **(E)** Heatmap shows the percentage of base incorporations opposite template adenines in the indicated contexts, highlighting the combined influence of nucleotides at both the -1 and -2 positions relative to adenine. **(F)** For each expected AAU, AAC, AAA, and AAG triplet within the *TR1*, *TR2*, and *TR3* templates, the corresponding observed triplets were identified in Illumina sequencing reads. The proportion of amino acids encoded by these observed triplets was calculated for each anticipated triplet and visualized as a heatmap. Heat maps were generated from 10 million subsampled reads pooled from all three *TR*s.

Our cDNA sequencing analysis confirms that misincorporation frequency and identity vary significantly depending on the adenine’s position within the RNA template (Fig 2G) [7]. While this suggests that the local sequence context directly influences error-prone reverse transcription, this relationship has remained systematically uncharacterized. To resolve this, we constructed a pDGR2 variant featuring a synthetic template encoding all 16 possible (NAN) trinucleotides within a single *TR*-RNA. To decouple contextual influence from a potential broader positional effect, we generated two more variants where the template was partitioned and rearranged, allowing us to evaluate how an adenine’s placement at the 5’, middle, or 3’ region of the RNA affects its mutagenic profile (Fig 3A). Following expression in *E. coli*, we deep-sequenced the resulting cDNAs to establish a profile of bRT’s context-dependent behavior.

This analysis revealed notable context-dependent effects on the mutagenic tendency of bRT that were reproducible across the three RNA templates (Fig 3A–3B), indicating that the sequences immediately adjacent to adenines are the primary determinants of the observed variations. However, our results diverged significantly from a prior *in vitro* finding. We measured an average misincorporation rate of 30.65% opposite all template adenines, which is significantly lower than the previously reported 51.2% rate (Fig 3B) [7]. Moreover, the error distributions also differ. In our study, cytosine (C) misincorporation predominated (59.3%), followed by guanosine (G, 26.4%) and adenine (A, 14.3%). It should be noted that the guanosine misincorporation rate is artificially inflated by a specific context with an unusual guanosine bias (see below); in most contexts, guanosine is disfavored. This contrasts with previously reported *in vitro* error profiles, which showed a significant preference for adenine misincorporation (A, 43% > C, 34.5% > G, 22.5%) [7].

While the non-uniform cellular dNTP pools—where dCTP is most abundant [15]—may partially account for bRT’s preference for cytosine misincorporations in our dataset, this factor is likely secondary to the intrinsic sequence context of the template. The natural BPP-1 template used in the *in vitro* study is heavily biased, with 20 of its mutable 23 adenines residing within an AAC context [1]. As demonstrated below, this unique error profile imposed by this specific motif can largely explain the discrepancies between *in vivo* and *in vitro* studies.

Our analysis clearly showed that bases immediately 3’ of template adenines (position -1) influenced the mutagenesis profile more profoundly than those immediately 5’ (position +1) (Fig 3C–3D). The most prominent example of this context dependence was seen with adenines adjacent a cytosine at the -1 position (Fig 3C). In this 5’-NAC-3’ context (where N is any base at position +1), guanosine (G) misincorporation was markedly elevated at 26.8%, contrasting sharply with the significantly lower rates observed in NAA (2.4%), NAG (0.08%) and NAT (2.5%) contexts (Fig 3C). This strong, context-dependent bias explains a previous observation: the template adenines found to induce unusually elevated G misincorporation in an earlier study [7] were, in fact, those residing in the same NAC context reported here. This preference for G was further magnified when a template adenine is flanked by two 3’ cytosines (ACC), where G misincorporation reached 44.3% (Fig 3E).

Interestingly, the influence of the 3’ cytosine in the AC context extends to the 5’ adenine one base upstream. In this AAC context, adenine misincorporation (23.8%), displacing cytosine (14.2%) as the most common error (Fig 3E). This effect was absent in closely related AAU, AAA, AAG motifs. Coupled with the heightened G misincorporation in the NAC context, the AAC motif exhibits a mutagenic profile distinct from any other adenine contexts. This finding is particularly noteworthy because the AAC motif is overly represented in native DGR templates, almost exclusively encoding for asparagine in-frame within the target gene [16].

Despite an overall 68% mutational rate, our analysis revealed that DGR-mediated mutagenesis of the AAC codon permits sampling of a highly restricted amino acid space. Only eight out of the 14 different accessible amino acids reached a frequency of at least 1% (Fig 3F), with the mutations dominated by tyrosine (14%), serine (12%) and aspartic acid (10%). Although the AAU codon theoretically explores the same amino acid space as AAC, the fact that cytosine (rather than adenine) is the most common misincorporation opposite the first template adenine biases mutations toward glycine and aspartic acid instead of the tyrosine favored by AAC. This divergence illustrates how context-dependent effects constrain the accessible evolutionary landscape.

Moreover, we observed that the ~ 1% conversion of AAA and AAG to stop codons (Fig 3F) falls significantly below the 6.36% and 7.08% respectively expected if the 30.65% misincorporation rate is equally distributed among G, C and A (see Methods). Thus, the bRT appears to have evolved an intrinsic misincorporation bias to not only favor certain amino acids but also minimize unfavorable premature truncation.

### Genetic screen identifies ExoI as an inhibitor of DGR activity

Having reconstituted DGR and characterized its mutagenic properties in *E. coli*, we next tried to improve upon its editing efficiency (~10^−5^), which remains too low for practical applications. We hypothesized that certain cellular factors antagonize DGR activity. To identify these potential inhibitors, we combined the kanamycin repair assay and transposon-sequencing (Tn-seq) to screen for genes whose inactivation enhance DGR activity [17].

**Fig 4:**
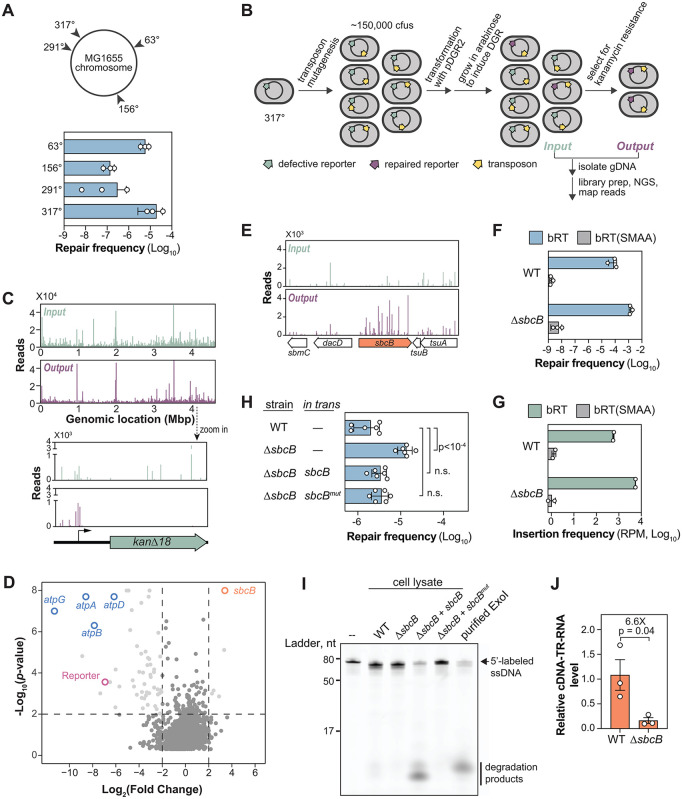
ExoI constrains DGR efficiency through a nuclease-independent mechanism. **(A)** Left, Chromosomal locations of reporter insertion. Right, Bar graph comparing the repair efficiency of reporters at the indicated chromosomal locations in MG1655. *E. coli* strains used in this experiment were: 63°(HCL34), 156°(HCL19), 291°(HCL24), and 317°(HCL26). **(B)** Schematic of Tn-seq workflow to identify factors affecting DGR-mediated reporter repair. See Methods for details. **(C)** Normalized Illumina read counts from each transposon insertion of the Input and Output libraries. Bump out shows changes in transposon distribution within the reporter cassette in the Input and Output libraries. **(D)** Volcano plot of transposon sequencing results. Vertical dotted lines represent thresholds for a four-fold increase or decrease in read counts. Data above the horizontal dotted line have Mann-Whitney *p*-value of 0.01 or less. **(E)** Normalized Illumina read counts from each transposon insertion surrounding the *sbcB* gene in the Input and Output libraries. **(F)** Bar graph showing the repair frequency of a chromosomal reporter in *E. coli* with the MG1655 (HCL26) vs ∆*sbcB* (HCL84) with and without a functional bRT. Error bars represent standard deviation of at least biological triplicates. bRT(SMAA) denotes catalytically inactive bRT expressed from a derivative of the pDGR2 plasmid. **(G)** Experiments were performed as in (**F**) except that reporter repair was read-out by sequencing reporters in the cell population without kanamycin selection. Shown are the frequency (in reads per million) of sequencing reads containing the expected 18 nucleotide insertion. n = 2 biological replicates. **(H)** Bar graph comparing the repair frequency of a chromosomally integrated reporter in MG1655 (WT) or ∆*sbcB* (HCL84) strain. These strains also contained either an empty vector pNEB309 (–), pNEB310 (expressing *sbcB*) or pNEB311 (expressing the nuclease-deficient *sbcB*^*Mut*^). **(I)** Cell-lysate-based ssDNA nuclease assay. A 5’ Alexa488 fluorescently labelled ssDNA (see S3 Table for sequence) was incubated for 10 minutes at room temperature with lysates prepared from strains used in **(H)** or commercially available purified ExoI (NEB MS293). These samples and the unreacted ssDNA substrate (–) were resolved on a 15% polyacrylamide gel before fluorescence imaging. **(J)** Bar graph showing the relative abundance of *TR*-RNA-cDNA hybrid in MG1655 vs ∆*sbcB* (HCL84) strain as measured by qRT-PCR. Error bars: standard error of the mean (SEM). n = 3 biological replicates. p-value was calculated by unpaired, two-tailed t-test.

To streamline the screen, we integrated the reporter cassette directly into the *E. coli* genome. Among the four tested chromosomal loci (Fig 4A), we chose the 317° locus (strain HCL26) for our screen, as it exhibited the highest repair frequency. We then mutagenized HCL26 using a mariner-based transposon harboring the chloramphenicol resistance gene for selecting cells with transposon insertions. We pooled ~150,000 chloramphenicol-resistant clones (Fig 4B) and transformed this mutant library with pDGR2 to initiate reporter repair. The library was then plated on kanamycin to select for cells where the reporter had been repaired by DGR. We deep-sequenced the transposon mutant library before (Input) and after kanamycin selection (Output) to identify enriched transposon mutants.

The result indicates that the screen identified genes altering the cell’s sensitivity to kanamycin as anticipated (Fig 4C–4D, S1 Table). Transposon insertions within the reporter cassette (Fig 4C) and genes coding for ATP synthase subunits (Fig 4D, S5 Fig) were severely depleted after selection. Inactivation of ATP synthase enhances the proton motive force (PMF), which consequently enhances kanamycin uptake [18–20]. This increases the cell’s antibiotic burden, causing impaired growth. This indicates that other hits in this screen, including many with metabolic function, may affect kanamycin sensitivity.

Despite this confounding factor, *sbcB* emerged as a promising inhibitor of DGR activity. In our screen, transposon insertions in the *sbcB* gene were enriched 10.5-fold in the Output relative to the Input (Fig 4D–4E). We validated this result by deleting the *sbcB* gene from the HCL26 reporter strain, which triggered a 19-fold increase in repair efficiency (Fig 4F). Importantly, this enhancement is strictly dependent on an intact bRT (Fig 4F). While *sbcB* mutations are known to activate a recombination pathway involving RecF under certain genetic backgrounds [21], the effect observed here is entirely independent of RecF (S6 Fig).

To rigorously exclude the possibility that *sbcB* deletion merely confers enhanced kanamycin tolerance, we used an orthogonal, antibiotic-independent assay to quantify DGR-mediated target editing. We deep-sequenced the reporter cassette isolated from the whole culture following induction, bypassing kanamycin selection entirely. Reporters isolated from the Δ*sbcB* background were found to be 10-fold more likely to have acquired the 18-nucleotide insert provided by the *TR* template compared to those from the wild-type strain (Fig 4G). Notably, both deep-sequencing and kanamycin repair assays yielded editing frequencies of the same order of magnitude. This high degree of quantitative consistency confirms that both methods accurately report the same underlying DGR activity, establishing that the observed enhancement reflects a genuine increase in mutagenic retrohoming.

The *sbcB* gene codes for Exonuclease I (ExoI), a processive 3’ to 5’ single-stranded DNA nuclease. Given that ExoI elimination enhances recombineering efficiency, presumably by stabilizing the ssDNA oligos [22], we reasoned that ExoI might similarly inhibit DGR by degrading the cDNA intermediate. To test this, we generated a nuclease-defective variant ExoI^Mut^, by substituting two conserved catalytic residues (D15 and E17) with alanines. Surprisingly, expression of ExoI^Mut^ *in trans* suppressed the elevated repair frequency in the Δ*sbcB* background as effectively as the wild-type nuclease (Fig 4H).

This unexpected result implies that the nuclease activity of ExoI is not required for inhibiting DGR. To confirm that ExoI^Mut^ was indeed nuclease-defective, we performed a cell-lysate-based nuclease assay [23]. As anticipated, lysates derived from *ΔsbcB* cells expressing wild-type ExoI rapidly degraded a labeled ssDNA substrate, whereas those expressing ExoI^Mut^ did not show this activity, confirming its nuclease deficiency (Fig 4I).

Direct quantification of the cDNA-*TR*-RNA—the presumptive substrate for ExoI—further contradicted a degradative role. Rather than being stabilized in the absence of the nuclease, the hybrid level was reduced by 6.6(± 3.0)-fold in the Δ*sbcB* background (Fig 4J). This paradoxical finding suggests that ExoI does not act by degrading the hybrid. Instead, the accumulation of hybrid in its presence suggests that it hinders downstream processing. Consequently, the rise in DGR efficiency upon sbcB deletion likely stems from the relief of this processing bottleneck, allowing the cDNA to be more efficiently channeled towards integration.

Additional evidence suggests how ExoI may hinder processing. We observed in the nuclease assay that overexpressing wild-type ExoI *in trans* triggered a massive increase in nuclease activity (Fig 4I, compare Lanes 2 and 4), yet this did not further reduce the repair frequency below that of the wild-type strain (Fig 4H). This saturation of the inhibitory effect, combined with the lack of nuclease dependence, suggests ExoI-mediated inhibition is not a result of cDNA degradation. Instead, it likely involves a stoichiometric interaction with a yet-to-be-identified partner, which caps the rate of DGR-mediated diversification.

### Chromosomal location and orientation of target genes affect DGR editing efficiency

**Fig 5:**
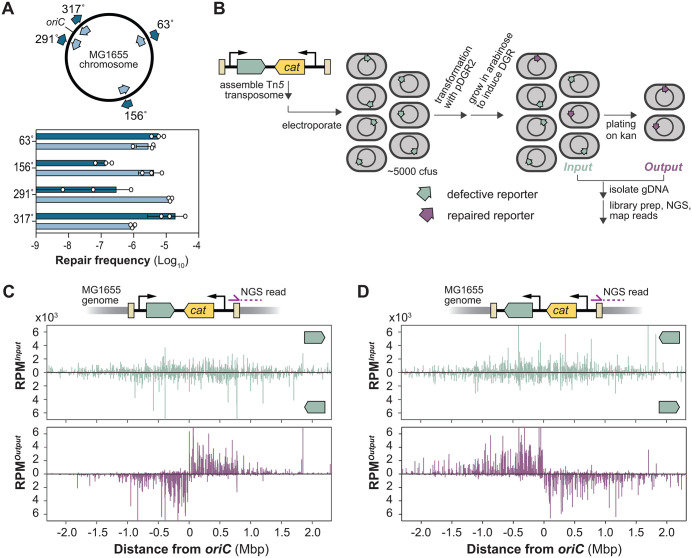
Chromosomal location and orientation of the target gene influence DGR activity. **(A)** Bar graph comparing the repair efficiency of reporters inserted at the indicated chromosomal locations in both orientations in *E. coli*. DGR components were expressed from pDGR2. n = 3; error bars indicate standard deviation. **(B)** Schematic of a high-throughput workflow for profiling the efficiency of DGR-dependent editing of thousands of chromosomally integrated reporters at once. **(C)** Schematic showing the reporter orientation within the transposon. Transposon sequencing workflow was used to map the right transposon-chromosome junction. In this transposon design, the reporter is co-directional with the sequencing read. Normalized Illumina read counts (reads per million, RPM) of mapped transposon insertion sites across the MG1655 genome for both the Input and Output libraries. Note that the genomic coordinate is adjusted to center the data around *oriC* (3,925,797 bp). **(D)** Same as described in **C**, except that the reporter orientation is reversed in the transposon used in this experiment. Consequently, the reporter orientation is contra-directional with the sequencing read. Data in **C** and **D**, are from a single experiment.

In Fig 4A, we observed a significant variation in the repair frequency among four genomic loci, differing by as much as 190-fold. To investigate whether target orientation also influences DGR editing, we constructed four more reporter strains, in which the orientation of the target at those chromosomal loci was reversed. Orientation reversal significantly affected repair frequency across all four loci, with the two reporters at the 291˚ locus showing the highest difference of 44-fold based on their orientation (Fig 5A). These results indicate that the efficiency of DGR editing depends on both the genomic location and orientation of the target.

To profile these effects at the genome scale, we used the Tn*5* transposase to introduce reporters at roughly 5000 chromosomal loci and used the Tn-seq workflow to measure their repair frequency in a single experiment (Fig 5B). Our analysis identified about 5000 unique target insertions throughout the chromosome in both orientations in the starting library (Fig 5C, top). However, the post-selection output library, which contained ‘repaired’ reporters, showed a dramatically different distribution (Fig 5C, bottom). This altered representation recapitulated the location and orientation bias seen in our low-throughput experiment (Fig 5A).

Reporters located near the origin of replication (*oriC*) became significantly overrepresented, indicating a higher repair frequency in this region. The higher copy number of targets near *oriC*, resulting from multi-fork replication in *E. coli* [24,25], likely explains the enhanced target editing efficiency in this region. Beyond this positional effect, we also observed a striking pattern dependent on reporter orientation (Fig 5C). Across the genome, one orientation is always preferred from the other, but the favored orientation abruptly switches at *oriC*. Specifically, reporters oriented leftward were preferentially enriched on the left side of *oriC*, while the opposite orientation (right-facing) was enriched on the right side of *oriC*. This pattern demonstrates that the DGR mechanism consistently prefers reporters that point away from *oriC*.

To confirm that the observed genome-wide trends were not an artifact of our transposon design, we reversed the reporter orientation in a new transposon. This control resulted in a corresponding reversal in the enriched transposon orientation (Fig 5D). Thus, DGR editing efficiency is inherently influenced by reporter orientation, and the preferred orientation is dictated by the target’s genomic position relative to *oriC*. This finding suggests a programmable mechanism for using a single DGR to diversify multiple targets at differential rates within the same cell.

### Transcription cannot explain the orientation bias

We next sought to understand why target genes pointing away from *oriC* exhibited higher DGR editing efficiency. Bacterial chromosomes typically orient highly transcribed genes co-directionally with DNA replication to prevent genome instability arising from head-on collisions between the RNA polymerase and the replisome [26]. We therefore hypothesized that the directional enrichment of stronger promoters could indirectly enhance the transcriptional output of reporters oriented away from *oriC*, thereby increasing their DGR editing efficiency. The kanamycin reporter assay is ill-suited to investigate this hypothesis, as altering the expression of the reporter would confound measurements of resistance. Instead, we used sequencing to measure the rate of A-to-N mutations directly as a function of the target’s promoter strengths.

**Fig 6:**
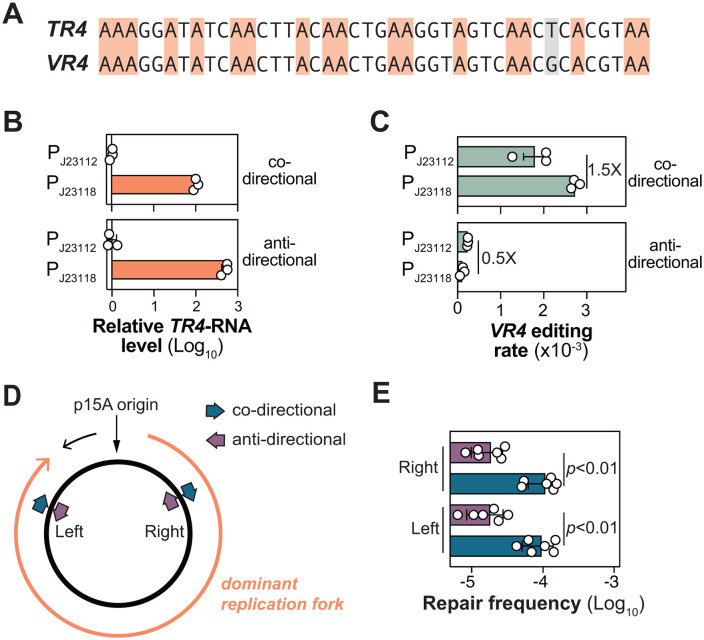
Target orientation relative to replication directionality determines DGR efficiency. **(A)** Sequences of *TR4* and *VR4* used for deep sequencing analysis of DGR-mediated adenine mutations. Co-transfer of T from *TR4* to replace G in *VR4* (colored grey) was used as a signature to distinguish DGR-induced adenine mutations from spontaneous adenine mutations. **(B)** qPCR analysis of reporter expression levels in *E. coli* MG1655. Total RNA was extracted from cells harvested at OD_600_ ~ 0.6. The *VR4* reporter, controlled by a weak (P_J23112_) or strong (P_J23118_) constitutive promoter, was integrated at the 291˚ locus in a derivative of MG1655 strain lacking Δ*sbcB* Δ*mutS*. Reporters were inserted in either co-directional or anti-directional orientations with replication, generating the strains HCL124 (P_J23112_, co-directional), HCL121 (P_J23112_, anti-directional), HCL126 (P_J23118_, co-directional) and HCL123 (P_J23118_, anti-directional). For each orientation, reporter expression levels were normalized to *tufA* and then to the expression level of the P_J23112_. Error bars denote the standard error of the mean for three biological replicates. **(C)** Deep-sequencing quantification of DGR-mediated editing rates of the *VRs* described in **A**. Cells of the indicated *E. coli* strains were grown in LB + 0.2% arabinose until OD_600_ ~ 2. Genomic DNA was isolated from harvested cells, and the *VR* regions were PCR amplified for sequencing. The mutagenesis rate of *VR4* represents the fraction of sequencing reads with both at least one A-to-N mutation and the designed G-to-T mutation in the *TR4* region and then dividing it to the total number of reads in that sample. Error bars denote the standard error of the mean for three biological replicates. Regardless of the transcriptional output, opposing reporters showed significantly different mutational rates: p-values are 0.004 (P_J23112_) and <0.0001 (P_J23112_). **(D)** Schematic of a plasmid with a p15A origin of replication. The dominant replication fork replicates almost of the entire plasmid (marked by orange arrow). Reporters were inserted in both co-directional and anti-directional orientations relative to the dominant replication fork at the indicated sites, creating pNEB298 (right, anti-directional), pNEB299 (right, co-directional), pNEB300 (left, anti-directional), and pNEB301 (left, co-directional). **(E)** Repair frequency of reporters described in **D**. Measurements were performed in the *E. coli* MG1655 strain using the kanamycin repair assay, with each p15A-based plasmid co-transformed with pDGR2. Bar heights are the mean of n = 6 biological replicates; error bars indicate the standard deviation.

For this assay, we designed a new reporter (*VR4*) with 18 adenines and drove its expression with either a weak (P_J23112_) or strong (P_J23118_) constitutive synthetic promoters (Fig 6A). To maximize mutation detection efficiency, we strategically incorporated three key design features learned from our study: we included many highly mutagenic AAC (Fig 3E); we used the highly active Δ*sbcB* strain (Fig 4); and we inserted the reporter near *oriC* (291°) (Fig 5C–5D). We also inactivated DNA mismatch repair by deleting *mutS*. While the 18-nt insertion is not a substrate for MutS in our kanamycin repair assay (where it showed no effect, S7 Fig) [27], deleting *mutS* should help preserve any DNA substitutions introduced by DGR in this assay. Finally, to control against spontaneous adenine mutation, we introduced a G-to-T signature mutation into the template (*TR4*; Fig 6A shaded grey), which was otherwise identical in sequence to *VR4*. The co-transfer of this mutation alongside the adenine mutations allowed us to definitively identify DGR-specific mutations.

After confirming that these promoters exhibited the expected difference in transcriptional output (Fig 6B), we transformed these strains with a pDGR2 variant to express the corresponding *TR4-*RNA alongside bRT and Avd. We then sequenced the reporter to quantify the rate of A-to-N mutations. Consistent with our previous result (Fig 3E), adenines in the AAC context exhibited significantly higher mutations rates (S8 Fig). Furthermore, targets oriented co-directionally with replication accrued mutations at a higher rate than those in the reverse orientation (weak promoter: 7.6-fold, strong promoter: 24.6-fold) (Fig 6C). However, for reporters with the same orientation, an orders-of-magnitude difference in transcriptional output only mildly affected the rate of A-to-N mutations (Fig 6C). Thus, while transcription contributes to mutagenic retrohoming, it cannot explain the observed orientation effects.

### Replication directionality dictates the preferred target orientation

As with most bacteria, chromosome replication in *E. coli*, initiates at the *oriC*, forming two replication forks that migrate in opposite directions [25,28]. This bifurcating replication direction aligns precisely with the chromosomal reporter orientations that favor DGR editing (Fig 5C–5D). To test whether DNA replication direction causes this target orientation bias, we measured the repair frequency of reporters encoded on a plasmid with a p15A origin. In contrast to bidirectional chromosomal replication, p15A-based plasmids are replicated largely in one direction by a dominant replication fork [29] (Fig 6D). Unlike what was observed for chromosomal targets, a single reporter orientation exhibited consistently higher repair frequency, regardless of the reporter’s location with respect to the origin (Fig 6E). Crucially, the preferred target orientation on this plasmid aligned with the direction of the dominant replication fork, thus demonstrating that replication directionality dictates the preferred editing orientation.

We next sought to understand why targets co-directional with replication exhibit much higher DGR editing efficiency than those in the reverse orientation. We hypothesized that this effect stems from the inherent asymmetry in which the two complementary DNA strands are replicated. While the leading strand is replicated continuously with little exposed ssDNA region, the lagging strand is replicated in short, disjointed segments, separated by large ssDNA gaps [30]. This is significant because, in the preferred (co-directional) target orientation, the sequence complementary to the cDNA is located on the lagging-strand template (S9A Fig). Therefore, the higher efficiency may be attributed to enhanced cDNA homing into the extended single-strand gap.

To directly test this model, we attempted to narrow the size of the lagging-strand ssDNA gap by overexpressing the primase DnaG, expecting a corresponding reduction in lagging-strand editing efficiency. We observed no change in the reporter repair frequency (S9B Fig). We also measured DGR-mediated editing in a reporter strain that bears the dnaG(K580A) allele. The K580A mutation is known to diminish recruitment of DnaG to the site of lagging strand synthesis and therefore increase the size of the ssDNA gap [31]. However, the reporter repair frequency was unaffected by this genetic alteration (S9C Fig).

We consider these negative results inconclusive. The cDNA (~70 nucleotides) in our reporter assay is much smaller than the typical ~1–2 kilobase ssDNA gap [30]. It is still uncertain whether our genetic manipulations altered this gap sufficiently and reproducibly to yield a detectable change in DGR efficiency. Nonetheless, our data clearly implicate DNA replication as a major driving force in DGR efficiency, even as more sensitive assays remain needed to clarify the precise underlying mechanism.

## Discussion

We demonstrated for the first time that the entire mutagenic retrohoming pathway, spanning from mutagenic cDNA synthesis to target mutagenesis, can be reconstituted in a heterologous bacterial host. This was achieved by simply transplanting the four core components of the BPP-1 DGR (Fig 2). This result indicates that, at least for this specific DGR, any additional host factors required to promote mutagenic retrohoming are already present in *E. coli* and likely ubiquitous among many bacteria species. We leveraged the tractability and available tools of *E. coli* to comprehensively characterize and mechanistically dissect this reconstituted DGR.

We generated a reference map for bRT’s mutational rates at template adenines surrounded by diverse sequence contexts (Fig 3). Beyond guiding synthetic template design, this map offers new biochemical insights into the error-prone behavior of bRT within the cellular environment. A particularly prominent finding is the elevated guanosine misincorporation within AC and ACC contexts. This bias is remarkable (Fig 3E), as it occurs despite dCTP being six-fold more abundant than dGTP in *E. coli* [15]. This heightened error rate suggests a mechanism that transcends simple competition kinetics. We propose that this preference for guanosine stems from a temporary template slippage event. In this model, the elongating end of cDNA may temporarily lose register, causing the bRT to re-use the preceding cytosine to template the incoming nucleotide addition. Guanosine misincorporation is likely exacerbated in the ACC context (A in the 0 position) because slippage of the terminal guanosine of the cDNA is stabilized through base pairing with the template cytosine in the -2 position. To avoid insertions, however, register must somehow be restored before elongation continues. A similar model, involving slippage in multiple of three base pairs, was previously posited to explain the insertion or deletion events that sometimes occur within AAC repeats [16], suggesting that template slippage might be a key mechanism underlying bRT’s error profile. Obtaining a structural model of the reverse transcriptase complex poised to decode the ACC motif should help clarify the molecular basis of this phenomenon.

In systems that have been studied thus far, the main purpose of DGRs is to generate a diverse repertoire of antigen receptors, allowing organisms to adapt to changing antigens [1,32,33]. Evolution appears to have optimized DGRs to excel at this role through a multi-layered strategic control over the diversification process. The first strategy lies in the choice of the AAC and, to a lesser extent, AAU codons themselves. Mutating adenines within these codons (instead of AAA or AAG) enables the sampling of up to fifteen amino acids while avoiding the generation of stop codons, thus side-stepping the risk of protein truncation and loss of function. Second, amino acids encoded by AAC have evolved to cluster around antigen-binding surfaces. By concentrating diversification to only this region, DGRs can efficiently generate new binding characteristics without destabilizing the overall protein fold [32,34].

We have uncovered a potential third layer of strategy that helps DGRs to efficiently explore the astronomical repertoire of protein variants they can theoretically generate [2]. This strategy centers on the intrinsic bias of bRT (Fig 3A and 3E), which preferentially converts asparagines—encoded by the prevalent AAC motifs in DGR templates–into residues like tyrosine and serine (Fig 3F). These two amino acids are highly enriched in the antigen-binding surfaces of antibodies [35–37]. Tyrosine, in particular, is a privileged residue in molecular recognition due to its versatile physiochemical properties, while small, flexible amino acids like serine (and glycine) provide the structural plasticity necessary for contouring the antigen surface [35,36,38]. By prioritizing tyrosine while simultaneously disfavoring residues like arginine, which are notorious for promoting non-specific, promiscuous binding, bRT ensures a higher “hit rate” for functional binders. It is also tempting to speculate that the placement of AAC—and the occasional inclusion of non-AAC codons—within the target gene is evolutionarily mapped to calibrate the diversity of amino acid sampling at key functional locations (Fig 3F). Ultimately, by constraining changes to the “right places” and favoring the “right residues”, DGR transforms a random search into a targeted, accelerated exploration. Thus, DGRs function as a well-programmed diversity generator, ideally optimized for evolving a functional receptor within a viable biological timeframe.

Our findings also illuminate the interaction between DGRs and endogenous cellular factors. We found that the ExoI nuclease restricts DGR efficiency (Fig 4). Unexpectedly, this inhibitory effect is independent of ExoI’s nuclease function (Fig 4H–4I). Although such non-catalytic functions have been well-documented for this enzyme [21,23,39–41], the lack of a detailed mechanistic framework for these cases offers few clues into the nature of this inhibition. Our data support a model where inhibition occurs through a binding-mediated mechanism. This inhibitory function is retained in the nuclease-dead mutant. Interestingly, relief of this inhibition leads to the accumulation of cDNA-*TR*-RNA hybrids (Fig 4J). Naorem *et al.* previously proposed that this hybrid is processed to form an Okazaki fragment-like intermediate to facilitate target incorporation [6]. While a causal link remains to be established, our results are consistent with a model where ExoI binding sterically hinders this critical processing step. Although the binding partner remains unknown, the 3’ end of cDNA is an appealing candidate, as nuclease-deficient ExoI variants like Sbc15 retain a strong affinity for this terminus [42]. Alternatively, ExoI may sequester an entirely different host factor that is responsible for hybrid processing. Future research testing these models will be critical for elucidating how this physical interaction hinders the mutagenic retrohoming process.

We also discovered an intimate connection between DGRs and DNA replication (Figs 5 and 6). Specifically, on both the genome and plasmids, DGR efficiency is markedly enhanced when the target is oriented in the same direction as DNA replication. This finding provides important clues into the still mysterious process by which the mutagenized cDNA is integrated into the target gene. In the favored orientation, the target sequence complementary to the cDNA is replicated by lagging-strand synthesis. Consequently, this target sequence exists in a single-stranded state for a longer duration than the opposite strand. We interpret this as evidence that the cDNA exploits this transient window of increased accessibility to base pair with the target. The stem-loop-forming inverted repeat (Fig 2C) likely enhances this process by structurally prolonging the open state of the target sequence. By hindering immediate re-annealing, these structures may extend the temporal opportunity for cDNA integration.

While this model still awaits further experimental validation, our results suggest that lagging-strand synthesis is likely not the only process exploited by DGR for cDNA insertion. DGR target editing persists, albeit at a reduced efficiency, even when the target sequence lies on the leading-strand template (Figs 5A and 6E). Because ssDNA is virtually non-existent during leading-strand replication [43], other cellular processes that cause transient target unwinding—such as transcription—likely also allows cDNA integration (Fig 6C). This relative flexibility contrasts with some other mobile genetic elements that strictly rely on hijacking lagging-strand synthesis for insertion or excision [44].

**Fig 7:**
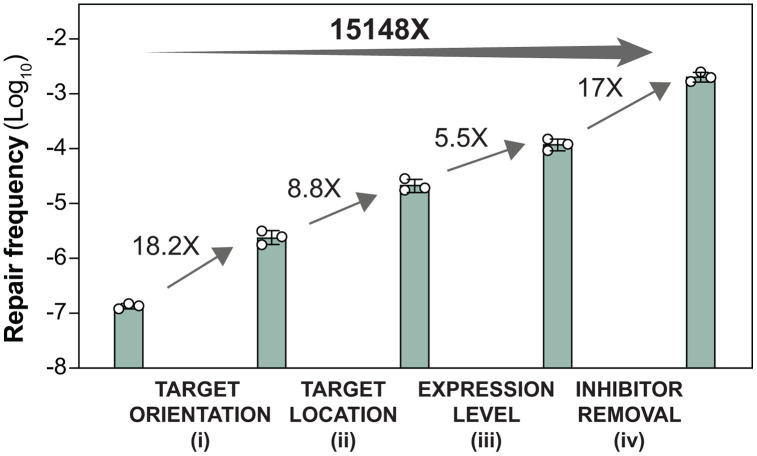
Molecular ‘knobs’ for fine-tuning DGR efficiency over 15,000-fold. Kanamycin repair assay was performed as described previously (Fig 2). Stepwise enhancement of DGR activity was achieved by i) aligning the target gene orientation with DNA replication, ii) moving the target closer to replication origin, iii) optimizing the expression of DGR components, and iv) removing the ExoI inhibitor. Relevant reporter strains and pDGR plasmids (arranged from left to right) were: HCL19/pDGR1, HCL20/pDGR1, HCL26/pDGR1, HCL26/pDGR2 and HCL84/pDGR2. Data shown here are from independent biological replicates performed separately from those presented in Figs 2, 4, and 5. Bar heights represent the mean of n = 3 biological replicates; error bars indicate the standard deviation.

The innate capacity of DGRs for massive, targeted diversification presents a tantalizing platform for diverse biotechnological applications [45]. However, the utility of such a system is contingent upon our ability to precisely tune its output. A key insight from our work is the identification of a set of discrete molecular ‘knobs’ that govern retrohoming efficiency. By systematically manipulating target location and orientation, modulating the expression of DGR components, and relieving an inhibitory mechanism via ExoI removal, we demonstrate that retrohoming activity in *E. coli* can be tuned across a remarkable 10^5^ range (Fig 7).

While the specific details may vary across species, the mechanistic logic underlying these molecular ‘knobs’ likely remains broadly conserved. For instance, although a clear ExoI homolog is absent in *Bordetella*, it is probable that DGR activity is constrained by other cellular factors whose removal would similarly enhance efficiency. We suspect that nature exploits these regulatory handles to calibrate DGR activity in accordance with specific physiological demands. One can imagine a scenario where DGR expression is coupled to stress-response pathways to accelerate adaptation during periods of cellular duress, or where the BPP-1 DGR harnesses the heightened replicative activity of the lytic phase to maximize host receptor diversification. Finally, DGRs acting on multiple targets [12] may leverage the genomic context of each target to impose varying diversification rates, thereby steering distinct evolutionary trajectories within a single cellular compartment. Elucidating the myriad ways in which DGR activity is regulated in the wild represents an exciting frontier.

Moving forward, we anticipate the strategies and mechanistic findings provided here will pave the way for a more complete mechanistic understanding of DGR and guide the rational engineering of these systems for biotechnological applications. Moreover, our work established a blueprint for using *E. coli* as a versatile heterologous host to investigate novel DGRs, particularly those from non-tractable organisms or harboring alternative accessory factors [46].

## Materials and methods

### Plasmid construction

Plasmids were either constructed by GenScript Biotech or generated in-house using NEBuilder HiFi DNA Assembly (NEB, E2621) or NEB KLD Enzyme Mix (NEB, M0554). The sequences of all plasmids used in this study are listed in S2 Table.

### Strains and growth conditions

All bacterial strains used in this study are listed in S3 Table. With few exceptions, all the reported experiments were performed in *E*. *coli* strain MG1655 and its derivatives. Cells were grown in grown in lysogeny broth (LB; 1% tryptone, 1% NaCl, 0.5% yeast extract; Teknova, L8000) or Lennox LB (1% tryptone, 0.5% NaCl, 0.5% yeast extract; Teknova, L5000) as indicated. Plasmids were maintained in NEB5α, except for those with an R6K origin, which were maintained in One Shot PIR2 (Invitrogen, C111110). Whenever appropriate, the following antibiotics were used: 100 μg/mL ampicillin (Amp100), 15 μg/mL chloramphenicol (Cm15), 25 μg/mL kanamycin (Kan25), 50 μg/ml Spectinomycin (Spec50), and 10 μg/mL tetracycline (Tet10).

### *E. coli* strain construction

*Reporter insertion: E. coli* strains with genomically integrated reporters were constructed by amplifying desired sequences from synthesized gene fragments ordered from IDT or Twist. The primers used for this amplification had 40–60 nucleotides of complementarity to the intended genomic insertion site. The purified PCR products were then electroporated into electrocompetent MG1655 cells that contained the Lambda Red recombineering plasmid pKD46. After a one-hour recovery in SOC (NEB, B9020), the cells were plated on the appropriate selective media to isolate single recombinants.

*Gene deletion:* Mutations from relevant strains in the KEIO *E. coli* knockout collection (Horizon Discovery) [47] were first verified by PCR, as described by Datsenko et al. [48]. The verified mutations were then transferred into an *E. coli* MG1655 background using P1 phage transduction [49].

To remove selectable markers flanked by FRT sites, the strain was transformed with pCP20 [50]. Final insertions were verified by PCR. All primers used in this study are listed in S4 Table.

### Kanamycin repair assay

3-5 colonies were inoculated into 3 mL LB with the appropriate antibiotics in 24-well deep well round bottom plates and sealed with a breathable film. The cultures were shaken at 300 rpm at 37°C for 90 minutes. Arabinose was added to 0.2% final concentration and grown for another 4.5 hours. OD_600_ of the cultures was recorded (~ 2 in most genetic backgrounds). To select for kanamycin resistance, appropriate dilutions of cultures were plated on LB supplemented with Kan25. Colony forming units (CFU) were recorded after a 16-hour incubation at 37°C. To calculate repair frequency, calibrated CFU/mL was divided by the total cells plated based on OD_600_ of the culture.

To sequence the chromosomal reporter prior to kanamycin selection, 1 mL of culture was collected by centrifugation and genomic DNA was extracted using Monarch Spin gDNA extraction kit (NEB, T3010). 75 ng of genomic DNA per sample was PCR amplified with Q5 DNA Polymerase (NEB, M0491) using equimolar mixture of forward primers (oNEB-203, 204, 205, 206) and reverse primers (oNEB-170, 171, 172). PCR product was used as the template for a second PCR to add Illumina barcodes. The amplicons were cleaned up with 1.2X NEBNext DNA Sample Purification Beads and sequenced on Illumina iSeq 2x 150 paired end run.

To sequence the reporter following kanamycin repair assay, resistant colonies were pooled and pelleted. Plasmids were isolated using Monarch Plasmid Miniprep Kit (NEB, T1010) and used as the template for amplifying the reporter region on pTarget. PCR were carried out with Q5 DNA Polymerase (NEB, M0491) using an equimolar mixture of forward primers (oNEB-145, 170, 171, 172) and an equimolar mixture of reverse primers (oNEB-173, 174, 175, 176). The first PCR was used as the template for a second PCR to add Illumina barcodes. The amplicons were cleaned up with 1.2X NEBNext DNA Sample Purification Beads and sequenced on Illumina NextSeq 500 2 x 150 paired end run. For RNA extraction, cells were pelleted and stored at -80°C.

RNA Extraction, *TR* RNA-cDNA hybrid sequencing, and quantification with RT-qPCR.

Total RNA was extracted from log-phase *E. coli* with Monarch Total RNA Extraction Kit (NEB, T2010). The on-column DNase I treatment step was skipped to preserve the cDNA-*TR*-RNA hybrid.

*cDNA sequencing*: To sequence the cDNA within cDNA-*TR* hybrids, 1 μg of the extracted RNA was reverse transcribed using Induro RT (NEB, M0681) with primers that bind to the 3’ end of the cDNA. These primers were oNEB-344 (for pDGR1, Fig 2E–2G) and oNEB-356 (for pDGR2 variants, Fig 3). The resulting RT reactions then served as templates for a PCR amplification across the cDNA-*TR*-RNA junction using Q5 DNA Polymerase (NEB, M0492). For the pDGR1 construct, a mixture of forward primers (oNEB-319, 320, 321, 322) and a mixture of reverse primers (oNEB-535, 536, 537, 538) were used. For the pDGR2 variants, the same forward primer mixture was used, but with a different reverse primer mixture (oNEB-460, 461, 462, 463). A subsequent PCR step was performed to add barcodes for multiplexed sequencing.

*Quantitative PCR (qPCR) analyses*: For cDNA-*TR* RNA hybrid qPCRs; 2 µg of RNA (DNase I treatment skipped) was reverse transcribed with SuperScript IV (Invitrogen, 18090010) using a specific primer (oNEB-344) or random hexamers (NEB, S1230S) according to manufacturer’s protocol. RT reactions were diluted 1:10 and 5 µL of the dilutions were used as template in a 20 µL qPCR reaction with 250 nM of forward and reverse primers (oNEB-301 and oNEB-345 for the cDNA-TR RNA hybrid and oNEB-313 and oNEB-314 for *tufA* internal control) and 2X Luna Universal qPCR Master Mix (NEB, M3003). For *TR*-RNA qPCRs; 10 µg of the RNAs were treated with DNase I (NEB, M0303) according to manufacturer’s recommendations and purified using Monarch Spin RNA Cleanup Kit (NEB, T2030). RT and qPCR reactions were then performed as described above with specific primers for *TR*-RNA only (oNEB-343 for RT and oNEB-300 and oNEB-346 for qPCR). *tufA* expression level was used as the internal control. All reactions were performed on a Bio-Rad CFX96 system, and the average of three technical replicates was taken for each independent biological replicate.

### Data analysis for A-mutational rate

Illumina sequencing reads were aligned to the reference substrate using the minimap2 aligner with the -x sr parameter, optimized for short read mapping [51]. For each of the sixteen sequence contexts (NAN) within the reference substrate, the frequency of each nucleotide observed at the central position was recorded. The percentage of misincorporation at the central adenine was calculated and is presented as a heatmap. Following and preceding nucleotide heatmaps were generated similarly. For the weblogos, Illumina reads from the *VR*s of three biological reads were combined, the target region was trimmed with cutadapt, a custom python script was used to filter only the 36-nt region that corresponds to the *VR*. A random 10,000,000 reads were selected from the triplicate read pools for each *TR* and fed into WebLogo (version 3.7.12) [52,53] with -U ‘probability’ argument to show the probability on the y-axis instead of the default bits. To generate the amino acid sampling heatmaps, we used the same randomly selected pool of 10,000,000 combined triplicate reads. For each expected AAN triplet in the *TR*1, *TR*2, and *TR*3 templates, the corresponding observed triplets were identified. The data for all three templates was combined, and the proportion of amino acids encoded by these observed triplets was calculated for each anticipated triplet and visualized as a heatmap.

### Theoretical stop codon conversion rate calculation

The mutation probability for an adenine (A) residue is 0.3065. Under the assumption of equal misincorporation probabilities—causing A to G, C, or T mutations with equal frequency—each specific mutation occurs at a rate of approximately 0.1022 (0.3065 ÷ 3). Conversely, the probability of the adenine remaining unchanged is 0.6935.

Following this logic, the probability of an AAG codon mutating to the stop codon TAG is approximately 7.08% (0.1022 × 0.6935 × 1.0000). Given that the DGR mechanism can diversify AAA into all three termination codons (TAA, TAG, and TGA), we calculated the specific probability for each transition:

- **AAA** *→* **TAA:** ≈ 0.1022 × 0.6935 × 0.6935 ≈ 0.0491 or 4.91%
- **AAA** *→* **TAG:** ≈ 0.1022 × 0.6935 × 0.1022 ≈ 0.0072 or 0.72%
- **AAA** *→* **TGA:** ≈ 0.1022 × 0.1022 × 0.6935 ≈ 0.0072 or 0.72%.

Consequently, the aggregate probability of an AAA codon mutating to any stop codon is approximately 6.36%.

### Transposon sequencing

*Genetic screen for identifying genes influencing DGR activity* (Fig 4)*:* The plasmid pNEB99 was constructed to generate the transposon mutant library. This plasmid encodes a hyperactive Himar1 mariner transposase under the control of an arabinose-inducible promoter, along with a transposon containing a chloramphenicol resistance gene. The pNEB99 plasmid was introduced into the reporter strain HCL26 via puddle mating. Overnight cultures of the MFDpir donor strain with pNEB99 (in LB with 0.2 mM DAP and Amp100) and the HCL26 recipient strain (in LB) were mixed at a 4:1 ratio and pelleted. The pellet was resuspended, spotted on a Lennox LB agar plate topspread with 0.2 mM DAP, and incubated for 24 hours at room temperature.

To select for cells with transposon insertions, cells were scraped from the mating spot, resuspended in Lennox LB, and plated on Lennox LB agar plates supplemented with 0.2% glucose and Cm15. Approximately 120,000–180,000 clones were pooled and used to prepare electrocompetent cells. These cells were then transformed in duplicate with pNEB132 (pDGR2) and immediately recovered at 30°C in 25 mL of Lennox LB with Amp100. Spot dilutions estimated that about 20 million cells received the plasmid, which is 100 times the size of the starting library. When the OD_600_ reached 0.5, this culture was used to inoculate a 500 mL Lennox medium culture supplemented with Amp100 and 0.2% arabinose to induce DGR expression. After a 16-hour incubation at 30°C, 1.6 × 10⁹ cells were harvested by centrifugation and designated as the Input. Simultaneously, cells were plated on Lennox LB agar supplemented with Kan25 and incubated at 25°C for 48 hours. Approximately 4.8 million CFUs, or more than 26 times the size of the starting library, were pooled and designated as the Output.

*Profiling the effect of target gene location and orientation* (Fig 5): Two transposon versions (defective kanamycin reporter in either orientation) were created. They were generated by PCR amplification from plasmids pNEB34 and pNEB35 using Q5 DNA Polymerase (NEB, M0491) and primers oNEB-192 and oNEB-193 to add the inverted repeats. The amplicons were purified and incubated with EZ-Tn5 Transposase to assemble the transposomes according to the manufacturer’s protocol. These transposomes were then electroporated into HCL1 cells. Following a 90-minute recovery in SOC (NEB, B9020), the cultures were plated on LB supplemented with Cm15 and incubated overnight at 37°C. Approximately 5,000 transformants per condition were pooled.

The resulting reporter libraries were made electrocompetent and transformed with pDGR2. The transformants were selected by plating on LB with Tet10 at 37°C. About 1.5 million CFUs were scraped and resuspended in LB with Tet10 to prepare a starter culture. A 200 mL starter culture was inoculated at an initial OD_600_ of 0.05 and grown for 50 minutes at 37°C with shaking at 230 rpm. DGR activity was then induced by adding 0.2% arabinose, and the culture was grown for an additional 6 hours. A 20 mL sample from each culture was frozen as the Input population before kanamycin selection. To select for cells with repaired reporters, 2 × 10^10^ cells were plated on LB with Kan25 and incubated overnight at 37°C. Approximately 25 million kanamycin-resistant colonies were pooled and frozen as the Output population.

### Tn-seq library preparation and deep sequencing

Genomic DNA was extracted from cells with Monarch Genomic DNA Purification Kit (NEB, T3010). 5 μg of genomic DNA was sheared with Covaris ML230 instrument with these parameters: peak intensity power 260 W; duration 5 seconds; duty factor 6; cycles per burst 50; iterations 5; dithering Y = 3.0; speed = 20.0. The fragments were size selected with 0.6X NEBNext DNA Sample Purification Beads (NEB, E6178) to enrich for 1-1.5 kb DNA fragments. Purified DNA was end repaired with NEBNext End Repair Module (NEB, E6050) and cleaned up again with 0.6X NEBNext DNA Sample Purification Beads. Eluted DNA was treated with 10 units of Terminal Transferase (NEB, M0315) in a 50 mL reaction with 1 μL 9.5 mM dCTP, 0.5 mM ddCTP, 4 μL 10X TdT Buffer at 37°C for 30 minutes to add a poly-C tail to the DNA. Following heat inactivation at 75°C for 20 min, poly-C-tailed DNA was purified with 0.6X NEBNext DNA Sample Purification Beads. Purified DNA was used as the template to amplify the transposon-genome junction. 16 cycles of PCR were carried out with Q5 DNA Polymerase (NEB, M0491) using an equimolar mixture of oNEB-198, 199, 200, and 201 as forward primers and oNEB-202 as the reverse primer (S4 Table). PCR products were cleaned up with 0.7X NEBNext DNA Sample Purification Beads and eluted in 40 µL water. A second nested PCR was performed with 20 µL of the cleaned-up PCR products in 100 µL reaction with barcoded i5 and i7 primers for 14 cycles. Barcoded samples were combined and purified with 0.7X NEBNext DNA Sample Purification Beads. Libraries were then sequenced on Illumina NextSeq 500 1 x 150 single end run.

### Data analysis for transposon-based screens

Sequencing reads were analyzed using a previously described pipeline [54]. Briefly, Cutadapt (version 4.5) [55] was used to remove adaptors and trim low-quality sequences. Trimmed reads were mapped to HCL26 (NC_000913 with reporter inserted at 317°) using Bowtie 1.0.0 [56]. Fold change in normalized sequencing reads for a given gene in the Output versus Input library were calculated and statistical significance was determined by applying the Mann-Whitney U test. Genes that had at least 2-fold change and p-value less than 0.05 were deemed hits. Gene Ontology [57,58] was used to annotate gene function.

To analyze the effect of reporter location and orientation, NovaSeq Illumina reads were trimmed for poly G tails using fastp program with the –trim_poly_g parameter [59,60]. Reads with the expected transposon junction sequence (AAGAGACAG) were selected and trimmed of this sequence. Only reads at least 25 nt long after trimming were retained for analysis. The trimmed reads were then mapped to the *E. coli* strain MG1655 reference genome (GenBank NC_000913) using the minimap2 aligner with the -x sr parameter, optimized for short read mapping [51]. Only primary alignments with a mapping quality of 60 were considered. The first mapped position and reference strand were saved as CSV files and plotted.

### ExoI exonuclease assay

Cells harboring plasmids encoding ExoI variants were cultured in LB supplemented with 1 mM IPTG and (antibiotics) for 16 hours at 37°C. A total of 15 OD_600_ units of cells were collected and lysed using 0.75 mL of NEB Express *E. coli* Lysis Buffer (NEB, P8116) and rotated for 20 minutes at room temperature. Total cell lysates were clarified by centrifugation at 10000 x g for 10 minutes at 4°C. For the assay, 40 µL reactions containing 20 µL clarified cell lysates and 0.1 µM 5’ Alexa488-labeled ssDNA probe with the sequence Alexa488N/AAGGGCAGGCTGGGAAATAACGCATCGCCTTCTATCGCCTTCTTGACGAGTTCTTCTAATAACGGGGCGCGCGGCGTCTG (ordered from IDT) was prepared in 1X ExoI reaction buffer (NEB, MS0293) and incubated at room temperature for 10 minutes. A positive control was included by adding 40 units of purified, commercially available ExoI (NEB, MS0293) instead of the clarified cell lysate. Reactions were stopped with Novex 2X TBE-Urea Sample Buffer (ThermoFisher, LC6876) and the products were resolved on a 15% TBE-Urea gel (ThermoFisher, EC68855BOX). Gels were imaged using a Cytivia Amersham Typhoon in fluorescence mode with the Alexa488 filter.

### Quantification of the DGR mutational frequencies to understand the effect of transcription

Cultures of reporter strains (weak promoter: HCL121 and 124, strong promoter: HCL123 and 126) with chromosomally integrated *VR4* carrying the pNEB153 plasmid were grown in LB supplemented with Tet10 and Ara0.2 for 16 hours at 37°C. Cells were harvested by centrifugation, and genomic DNA was extracted from cell pellets using Monarch Spin gDNA Extraction kit (NEB, T3010) and used as the template to amplify *VR4* for deep sequencing. Twelve PCR cycles were carried out with Q5 DNA Polymerase (NEB, M0491), an equimolar mixture of forward primers (oNEB-203, 204, 205, 206) and an equimolar mixture of reverse primers (oNEB-528, 529, 530, 531) (S4 Table). A second nested PCR (6 cycles) was performed with 1 µL of the PCR products in 50 µL reaction with barcoded i5 and i7 primers (NEB, E6441A. Barcodes columns C6-D8). Barcoded samples were combined and purified using 1.2X NEBNext DNA Sample Purification Beads. Libraries were then sequenced on Illumina NovaSeq 2 x 150 paired end run. To confirm the promoter strengths, RNA was extracted from 1mL of log phase cells from each strain with Monarch Total RNA Extraction Kit (NEB, T2010). 1 μg of the extracted RNA was reverse transcribed using Induro RT (NEB, M0681) and random hexamers. RT reactions were then diluted 1:10 and 5 µL of the dilution was used in 20 µL qPCR reaction with 250 nM oNEB-573 and 250 nM oNEB-574 primers and 2X Luna Universal qPCR Master Mix (NEB, M3003). *tufA* expression level was used as an internal control.

### Computational analysis for mutational frequencies to understand the effect of transcription

Paired end reads were merged into single reads with BBMap bbmerge [61] to lower the sequencing error rates. Primer binding sites and any sequence before and after were trimmed from the merged reads with Cutadapt (version 4.5) [55] to obtain the *VR4* sequences with --discard-untrimmed argument to only include sequences containing primer binding sites. Reads that passed this filter were subjected to two analyses:

1) Quantify the fraction of *VR4* edited by DGR: A custom python script was written to identify *VR4* sequences containing both a G-to-T mutation 8 nucleotide 5’ of the GC14 site (designed to distinguish between DGR-mediated and nonspecific adenine mutations, see Fig 6C for details) and at least one A-to-N mutation. The number of reads satisfying these criteria was then normalized to the total read number and plotted for the different conditions (Fig 6E).
2) Determine the mutagenesis rate of *VR4* at the nucleotide level: *VR4* sequences considered to be edited by DGR (see above) were mapped to the unmodified *VR4* sequence using minimap2 (version 2.26) [51] with the --sr argument for short reads. Primary alignments were then selected and sorted using samtools (version 1.17) [62]. To detect mutations with location information, samtools mpileup was used with these arguments on sorted primary alignments: -d 0 --min-MQ 20 --min-BQ 30 --output-BP-5 --no-output-ins --no-output-ins --no-output-del --no-output-del --no-output-ends. The mutation counts per location were then normalized to total read counts and plotted for each condition through the *VR* sequence (S8 Fig).

## Supporting information

S1 Fig Plasmid pDGR2 expresses higher levels of both TR-RNA and bRT compared to pDGR1. (A) RT-qPCR showing the *TR*-RNA expression levels relative to *tufA* expression with DGR expression plasmids pDGR1 or pDGR2. Error bars denote the standard error of the mean (SEM) of three biological replicates. p-value was calculated by unpaired, two-tailed t-test. (B) Immunoblot analysis of bRT expression in cells transfected with ALFA tagged versions of pDGR1 or pDGR2. Fluorescently labeled anti-ALFA single-domain antibody was used to detect bRT fused with ALFA tag. RNAP was used as a loading control.
(EPS)

S2 Fig The RecA recombinase does not contribute to the repair of the reporter. Kanamycin repair assay was performed in MG1655 (WT) and ∆*recA* (HCL166) strains harboring both pTarget and pDGR1. Data represent the mean of n = 3 biological replicates. Error bars represent standard deviation.
(EPS)

S3 Fig Evidence for DGR-mediated target editing. (A) Bar plot showing the percentage of reporters with recovered missing nucleotides. To obtain this data, plasmids were isolated from pooled kanamycin-resistant clones generated in the kanamycin repair assay (as described in Fig 2B), and the *VR* region in the pTarget plasmid was amplified for deep sequencing. The experiment was performed in *E. coli* MG1655 co-transformed with pTarget and either pDGR1 or pDGR2 variants encoding either wild-type (WT) or inactive (SMAA) bRT. (B) The kanamycin repair assay was performed on *E. coli* MG1655 co-transformed with pTarget and pDGR1. A region encompassing the shown VR region of the pTarget plasmid was amplified by colony PCR from four individual kanamycin-resistant clones and then subjected to Sanger sequencing. The resulting Sanger traces show an 18-nucleotide insertion just upstream of the tandem stop codons. The heterogeneity evident in the traces beginning at the 5’ junction of the insertion site provides evidence for co-existence of repaired and unrepaired pTarget plasmids in a single kanamycin-resistant clone.
(EPS)

S4 Fig DGR-mediated editing does not inherently exclude adenine mutations in the coding region of the reporter. (A) To test if DGR-mediated editing is somehow restricted to non-coding regions, three synonymous mutations (indicated by arrows in (B)) were introduced into the *TR* of both pDGR1 and pDGR2, creating pDGR1’ and pDGR2’, respectively. The kanamycin repair assay was then performed in *E. coli* MG1655 co-transformed with pTarget and either pDGR1’/pDGR2 or their original counterparts. The introduction of these synonymous mutations did not affect the repair frequency. Data represent the mean of n = 3 biological replicates. Error bars represent standard deviation. (B) Deep-sequencing analysis of DGR-mediated mutations. Kanamycin-resistant clones harboring plasmids pTarget and either pDGR2 or pDGR2’ were pooled for plasmids isolation. The corresponding *VR* region was amplified and subjected to deep-sequencing. Shown weblogos were generated from sequencing reads that have recovered the missing 18 nucleotides. Note that, in both cases, adenines mutations were restricted to the stop codons.
(EPS)

S5 Fig Transposon insertions within the ATP synthase operon depleted after kanamycin selection. Normalized Illumina read counts from each transposon insertion surrounding the ATP synthase operon in the Input and Output libraries.
(EPS)

S6 Fig Increased repair frequency upon *sbcB* deletion is not due to activation of the RecF recombination pathway. Repair frequency of a chromosomal reporter in strain HCL26 (reporter integrated at 317˚) and its derivatives (∆*recF,* HCL158; ∆*sbcB,* HCL84; ∆*recF*∆*sbcB,* HCL162) harboring plasmid pDGR2. Data represent the mean of three biological replicates; error bars represent standard deviation.
(EPS)

S7 Fig The mismatch repair component MutS does not contribute to the repair of the reporter. Kanamycin repair assay if a chromosomal reporter was performed in MG1655 (WT HCL26) and *ΔmutS* (HCL95) strains harboring plasmid pDGR2. Data represent the mean of three biological replicates; error bars represent standard deviation.
(EPS)

S8 Fig Effects of promoter strength and gene orientation on the accumulation of adenine mutations on the *VR4* reporter. Shown are nucleotide resolution adenine mutagenesis rates from the same analyses as shown in Fig 6E. The data, organized by promoter strength (indicated in the top-left corner), are shown for the *VR4* reporter integrated in two orientations relative to chromosome replication: co-directional (A) and anti-directional (B). The designed G-to-T mutation, highlighted in gray, was used to distinguish DGR-mediated adenine mutations from background noise. Data represent the mean of n = 3 biological replicates. Error bars represent standard deviation. For details on the strains used, refer to Fig 6D.
(EPS)

S9 Fig Alteration of DnaG primase activity does not influence DGR-mediated reporter editing. (A) Schematic of a replication fork. The lagging strand template is shown to have a larger single-stranded DNA (ssDNA) region compared to the leading strand template, providing better access for DGR-generated cDNA to bind. (B) Overexpressing DnaG to reduce ssDNA gap size at the lagging strand had no measurable effect on the reporter’s repair frequency. The *E. coli* strain HCL26, which contains a defective kanamycin reporter inserted co-directionally with replication at the 317˚ locus, was co-transformed with pDGR2 and either an empty vector (pNEB309) or a plasmid expressing *dnaG* under an IPTG promoter (pNEB312). Kanamycin assay was carried out as described in Fig 2B, but with the addition of IPTG to the growth media. Note that IPTG addition reduced the overall editing efficiency by ~25-fold, likely due to IPTG interference of arabinose-inducible expression of DGR components [64]. (C) Bar graph showing that weakening of DnaG recruitment to the replication fork did not affect repair frequency. The K580A mutation in the native *dnaG* gene is known to weaken interaction with the replisome [31]. This mutation has been shown to increase recombineering efficiency, presumably due to increased size of lagging strand ssDNA region in *E. coli* [65]. A reporter strain harboring this *dnaG* mutation HCL136 did not alter the reporter repair frequency when compared to an otherwise isogenic control strain HCL26. Note that we were unsuccessful in generating a strain with the *dnaG*(Q576A) allele. Shown data represent the mean of n = 6 biological replicates. Error bars represent standard deviation.
(EPS)

S1 Table Tn-Seq hit list. (DOCX)

S2 Table Plasmids used in this study. (XLSX)

S3 Table Strains used in this study. (DOCX)

S4 Table Oligos used in this study. (DOCX)

S1 File Methods for supplemental figs. (DOCX)
