# Supplementary material for: High-throughput analyses of a reconstituted diversity-generating retroelement identify intrinsic and extrinsic determinants of diversification
Source: PLoS Genet. 2026 Feb 5;22(2):e1012038. doi: 10.1371/journal.pgen.1012038 (PMC12875486; doi:10.1371/journal.pgen.1012038)
Supplement: S1 Table — (DOCX) [file pgen.1012038.s010.docx]

| **Table S1: Tn-seq hit genes** | | |  |  |  |
| --- | --- | --- | --- | --- | --- |
| **Name** | **Length** | **Log_2_(Fold Change)** | **Mann  P-value** | **Function** | **Notes** |
| *atpG* | 863 | -11.29 | 0.000 | metabolism | ATP synthase |
| *sapC* | 890 | -8.90 | 0.000 | transporter | putrescine ABC exporter |
| *atpA* | 1541 | -8.59 | 0.000 | metabolism | ATP synthase |
| *atpB* | 815 | -7.86 | 0.000 | metabolism | ATP synthase |
| *clpP* | 623 | -7.33 | 0.001 | folding | protein degradation |
| *kanR* | 773 | -6.93 | 0.000 | antibiotic resistance | Experiment reporter |
| *gmB* | 575 | -6.51 | 0.000 | metabolism | ADP-heptose biosynthesis pathway |
| *atpD* | 1382 | -6.16 | 0.000 | metabolism | ATP synthase |
| *sdhD* | 347 | -6.04 | 0.005 | metabolism | succinate dehydrogenase |
| *sdhA* | 1766 | -6.02 | 0.000 | metabolism | succinate dehydrogenase |
| *sucC* | 1166 | -5.70 | 0.000 | metabolism | succinyl-CoA synthetase |
| *aceE* | 2663 | -5.07 | 0.000 | metabolism | pyruvate dehydrogenase |
| *ppk* | 2066 | -4.91 | 0.000 | metabolism | polyphosphate kinase |
| *wzxE* | 1250 | -4.76 | 0.000 | metabolism | lipid flippase |
| *guaB* | 1466 | -4.64 | 0.000 | metabolism | guanine biosynthesis |
| *araC* | 878 | -4.55 | 0.000 | DNA binding | transcription factor; side effect of our set up |
| *nuoB* | 662 | -4.44 | 0.000 | metabolism | quinone oxidoreductase |
| *ftsN* | 959 | -4.19 | 0.001 | metabolism | cell division protein |
| *apaH* | 842 | -4.18 | 0.000 | Antibiotic resistance | published [1, 2] |
| *pitA* | 1499 | -3.98 | 0.000 | transporter | metal phosphate H^+^ symporter |
| *trkH* | 1451 | -3.81 | 0.000 | transporter | potassium transporter |
| *dnaK* | 1916 | -3.81 | 0.000 | folding | chaperone protein |
| *nuoC* | 1790 | -3.76 | 0.002 | metabolism | quinone oxidoreductase |
| *purH* | 1589 | -3.75 | 0.001 | metabolism | purine biosynthesis |
| *hflC* | 1004 | -3.67 | 0.005 | metabolism | regulator of ftsH protease |
| *nuoG* | 2726 | -3.65 | 0.000 | metabolism | quinone oxidoreductase |
| *hflK* | 1259 | -3.56 | 0.002 | metabolism | regulator of ftsH protease |
| *sucD* | 869 | -3.53 | 0.000 | metabolism | succinyl-CoA synthetase |
| *slt* | 1937 | -3.44 | 0.000 | metabolism | peptidoglycan recycling |
| *nuoL* | 1841 | -3.41 | 0.001 | metabolism | quinone oxidoreductase |
| *qseC* | 1349 | -3.33 | 0.001 | metabolism | quorum sensing |
| *clpX* | 1274 | -3.28 | 0.000 | folding | protein degradation |
| *purM* | 1037 | -3.24 | 0.004 | metabolism | purine biosynthesis |
| *sdhB* | 716 | -3.23 | 0.000 | metabolism | succinate dehydrogenase |
| *bipA* | 1823 | -3.18 | 0.000 | translation | 50S ribosomal subunit assembly factor |
| *tgt* | 1127 | -3.13 | 0.001 | translation | tRNA-guanine transglycosylase |
| *aspA* | 1436 | -3.12 | 0.000 | metabolism | aspartate ammonia lyase |
| *trkA* | 1376 | -3.10 | 0.000 | transporter | potassium transporter |
| *cyaA* | 2546 | -3.02 | 0.000 | metabolism | adenylate cyclase |
| *gltA* | 1283 | -3.02 | 0.000 | metabolism | citrate synthase |
| *hldE* | 1433 | -2.99 | 0.000 | metabolism | LPS biosynthesis |
| *rnb* | 1934 | -2.72 | 0.000 | nuclease | RNAse II works on tRNA |
| *purL* | 3887 | -2.70 | 0.000 | metabolism | purine biosynthesis |
| *yjgA* | 551 | -2.70 | 0.001 | translation | association with 23S rRNA |
| *gor* | 1352 | -2.61 | 0.004 | metabolism | glutathione reductase |
| *ptsN* | 491 | -2.48 | 0.006 | metabolism | phosphotransferase system enzyme IIA |
| *epmB* | 1028 | -2.40 | 0.001 | metabolism | lysine aminomutase |
| *galU* | 908 | -2.37 | 0.001 | metabolism | galactose breakdown |
| *polA* | 2786 | -2.34 | 0.000 | DNA binding | DNA polymerase I |
| *fabF* | 1241 | -2.23 | 0.001 | metabolism | fatty acid biosynthesis |
| *srmB* | 1334 | -2.20 | 0.009 | translation | RNA helicase associated with assembly of the ribosome |
| *acnB* | 2597 | -2.18 | 0.000 | metabolism | citrate to isocitrate |
| *tolC* | 1481 | -2.12 | 0.001 | transporter | known efflux pump for antibiotics |
| *relA* | 2234 | -2.07 | 0.001 | antibiotic resistance | published [3] |
| *fliM* | 1004 | 2.28 | 0.001 | motility | flagella |
| *narL* | 650 | 2.42 | 0.001 | DNA binding | transcription factor |
| *greA* | 476 | 2.89 | 0.001 | transcription | elongation factor |
| *dusB* | 965 | 3.33 | 0.000 | translation | tRNA function |
| *sbcB* | 1427 | 3.39 | 0.000 | Validation | exonuclease |

**REFERENCES**

1. Ji X, Yu R, Zhu M, Zhang C, Zhou L, Cai T, et al. Diadenosine tetraphosphate modulated quorum sensing in bacteria treated with kanamycin. BMC Microbiol. 2023;23(1):353. Epub 20231117. doi: 10.1186/s12866-023-03113-3. PubMed PMID: 37978430; PubMed Central PMCID: PMCPMC10657157.

2. Ji X, Zou J, Peng H, Stolle AS, Xie R, Zhang H, et al. Alarmone Ap4A is elevated by aminoglycoside antibiotics and enhances their bactericidal activity. Proc Natl Acad Sci U S A. 2019;116(19):9578-85. Epub 20190419. doi: 10.1073/pnas.1822026116. PubMed PMID: 31004054; PubMed Central PMCID: PMCPMC6511005.

3. Knutsson Jenvert RM, Holmberg Schiavone L. Characterization of the tRNA and ribosome-dependent pppGpp-synthesis by recombinant stringent factor from Escherichia coli. FEBS J. 2005;272(3):685-95. doi: 10.1111/j.1742-4658.2004.04502.x. PubMed PMID: 15670150.
