# Supplementary material for: High-throughput analyses of a reconstituted diversity-generating retroelement identify intrinsic and extrinsic determinants of diversification
Source: PLoS Genet. 2026 Feb 5;22(2):e1012038. doi: 10.1371/journal.pgen.1012038 (PMC12875486; doi:10.1371/journal.pgen.1012038)
Supplement: S3 Table — (DOCX) [file pgen.1012038.s012.docx]

**Table S3: Strains used in this study**

| **ID** | **Genotype** | **Construction/Source^a,b^** |
| --- | --- | --- |
| HCL1 | K-12 F– λ– ilvG– rfb-50 rph-1 | Laboratory strain of *Escherichia coli* K-12 (MG1655) [1] |
| HCL19 | HCL1 156˚::kan∆18(+) | DGR kan∆18 reporter inserted on the plus strand at 156 degrees using recombineering |
| HCL20 | HCL1 156˚::kan∆18(-) | DGR kan∆18 reporter inserted on the minus strand at 156 degrees using recombineering |
| HCL24 | HCL1 291˚::kan∆18(+) | DGR kan∆18 reporter inserted on the plus strand at 291 degrees using recombineering |
| HCL25 | HCL1 291˚::kan∆18(-) | DGR kan∆18 reporter inserted on the minus strand at 291 degrees using recombineering |
| HCL26 | HCL1 317˚::kan∆18(+) | DGR kan∆18 reporter inserted on the plus strand at 317 degrees using recombineering |
| HCL27 | HCL1 317˚::kan∆18(-) | DGR kan∆18 reporter inserted on the minus strand at 317 degrees using recombineering |
| HCL34 | HCL1 63˚::kan∆18(+) | DGR kan∆18 reporter inserted on the plus strand at 63 degrees using recombineering |
| HCL35 | HCL1 63˚::kan∆18(-) | DGR kan∆18 reporter inserted on the minus strand at 63 degrees using recombineering |
| HCL84 | HCL26 *sbcB*::*frt* | P1(KEIO *sbcB::kanR*) X HCL26 |
| HCL94 | HCL1 *sbcB::frt* | P1(KEIO *sbcB::kanR*) X HCL1 |
| HCL112 | HCL94 *mutS::frt* | P1(KEIO *mutS::kanR*) X HCL94 |
| HCL121 | HCL112 291˚::P_J23112_(+) | P_J23112_-*VR4* inserted on the plus strand at 291˚ using recombineering |
| HCL123 | HCL112 291˚::P_J23118_(+) | P_J23118_-*VR4* inserted on the plus strand at 291˚ using recombineering |
| HCL124 | HCL112 291˚::P_J23112_(-) | P_J23112_-*VR4* inserted on the minus strand at 291˚ using recombineering |
| HCL126 | HCL112 291˚::P_J23118_(-) | P_J23118_-*VR4* inserted on the minus strand at 291˚ using recombineering |
| HCL166 | HCL1 *recA::frt* | P1(KEIO *recA::kanR*) X HCL1 |
| HCL158 | HCL26 *recF::frt* | P1(KEIO *recF::kanR*) X HCL26 |
| HCL162 | HCL84 *recF::frt* | P1(KEIO *recF::kanR*) X HCL84 |
| HCL95 | HCL26 *mutS::frt* | P1(KEIO *mutS::kanR*) X HCL26 |
| HCL136 | HCL26 *dnaG::dnaG(K580A)* | See materials and methods |
| HCL4 | [ΔMu1::*aac(3)IV*-Δ*aphA*-Δ*nic*35-ΔMu2::*zeo*] Δ*dapA*::(*erm*-*pir*) Δ*recA* | Mating strain of *Escherichia coli* (MFD*pir*) [2] |

^a^ The *kanR* cassette from the KEIO library is flanked by *frt* sites allowing for its removal by FLP recombinase. An *frt* scar remains following removal.

^b^ P1 transduction denoted using the following shorthand: P1(donor) x recipient. Transductants were selected on LB + Kan plates.

**REFERENCES**

1. Blattner FR, Plunkett G, 3rd, Bloch CA, Perna NT, Burland V, Riley M, et al. The complete genome sequence of Escherichia coli K-12. Science. 1997;277(5331):1453-62. doi: 10.1126/science.277.5331.1453. PubMed PMID: 9278503.

2. Babic A, Guerout AM, Mazel D. Construction of an improved RP4 (RK2)-based conjugative system. Res Microbiol. 2008;159(7-8):545-9. Epub 20080627. doi: 10.1016/j.resmic.2008.06.004. PubMed PMID: 18638548.
