# Supplementary material for: High-throughput analyses of a reconstituted diversity-generating retroelement identify intrinsic and extrinsic determinants of diversification
Source: PLoS Genet. 2026 Feb 5;22(2):e1012038. doi: 10.1371/journal.pgen.1012038 (PMC12875486; doi:10.1371/journal.pgen.1012038)
Supplement: S4 Table — (DOCX) [file pgen.1012038.s013.docx]

**Table S4: Oligos used in this study**

| **Number** | **Sequence** | **Description** |
| --- | --- | --- |
| Sequencing Primers | | |
| oNEB-145 | CTTTCCCTACACGACGCTCTTCCGATCTVHDBGTATCGCCGCTCCCGATTC | p5, NGS primer for sequencing VR in kan reporter |
| oNEB-170 | CTTTCCCTACACGACGCTCTTCCGATCTHMGTATCGCCGCTCCCGATTC | p5, NGS primer for sequencing VR in kan reporter |
| oNEB-171 | CTTTCCCTACACGACGCTCTTCCGATCTNGTATCGCCGCTCCCGATTC | p5, NGS primer for sequencing VR in kan reporter |
| oNEB-172 | CTTTCCCTACACGACGCTCTTCCGATCTGTATCGCCGCTCCCGATTC | p5, NGS primer for sequencing VR in kan reporter |
| oNEB-173 | GAGTTCAGACGTGTGCTCTTCCGATCTBRRGKCTCAAATGCCTGAGGTTTTTAACG | p7, NGS primer for sequencing VR in kan reporter |
| oNEB-174 | GAGTTCAGACGTGTGCTCTTCCGATCTDRCTCAAATGCCTGAGGTTTTTAACG | p7, NGS primer for sequencing VR in kan reporter |
| oNEB-175 | GAGTTCAGACGTGTGCTCTTCCGATCTBCTCAAATGCCTGAGGTTTTTAACG | p7, NGS primer for sequencing VR in kan reporter |
| oNEB-176 | GAGTTCAGACGTGTGCTCTTCCGATCTCTCAAATGCCTGAGGTTTTTAACG | p7, NGS primer for sequencing VR in kan reporter |
| oNEB-198 | CTTTCCCTACACGACGCTCTTCCGATCTDKGSCATATGGACCATGGCTAATTCC | p5, NGS primer for sequencing the left transposon junction |
| oNEB-199 | CTTTCCCTACACGACGCTCTTCCGATCTDKCATATGGACCATGGCTAATTCC | p5, NGS primer for sequencing the left transposon junction |
| oNEB-200 | CTTTCCCTACACGACGCTCTTCCGATCTNCATATGGACCATGGCTAATTCC | p5, NGS primer for sequencing the left transposon junction |
| oNEB-201 | CTTTCCCTACACGACGCTCTTCCGATCTCATATGGACCATGGCTAATTCC | p5, NGS primer for sequencing the left transposon junction |
| oNEB-202 | GAGTTCAGACGTGTGCTCTTCCGATCTHGGGGGGGGGGGGGGGGH | p7, NGS primer for transposon sequencing, primes to polyC |
| oNEB-203 | GAGTTCAGACGTGTGCTCTTCCGATCTBYYGGTTCCATCGGTTGCCTTGG | p5, NGS primer for sequencing DGR reporter strength assay |
| oNEB-204 | GAGTTCAGACGTGTGCTCTTCCGATCTBTGGTTCCATCGGTTGCCTTGG | p5, NGS primer for sequencing DGR reporter strength assay |
| oNEB-205 | GAGTTCAGACGTGTGCTCTTCCGATCTBGGTTCCATCGGTTGCCTTGG | p5, NGS primer for sequencing DGR reporter strength assay |
| oNEB-206 | GAGTTCAGACGTGTGCTCTTCCGATCTGGTTCCATCGGTTGCCTTGG | p5, NGS primer for sequencing DGR reporter strength assay |
| oNEB-319 | GAGTTCAGACGTGTGCTCTTCCGATCTBYYCCTTCTTGCATGGCTCTGC | p7, NGS primer for sequencing cDNA-TR RNA hybrid for A mutagenesis assay |
| oNEB-320 | GAGTTCAGACGTGTGCTCTTCCGATCTBTCCTTCTTGCATGGCTCTGC | p7, NGS primer for sequencing cDNA-TR RNA hybrid for A mutagenesis assay |
| oNEB-321 | GAGTTCAGACGTGTGCTCTTCCGATCTBCCTTCTTGCATGGCTCTGC | p7, NGS primer for sequencing cDNA-TR RNA hybrid for A mutagenesis assay |
| oNEB-322 | GAGTTCAGACGTGTGCTCTTCCGATCTCCTTCTTGCATGGCTCTGC | p7, NGS primer for sequencing cDNA-TR RNA hybrid for A mutagenesis assay |
| oNEB-460 | CTTTCCCTACACGACGCTCTTCCGATCTVHDBCTGCGTCCCTGTGGC | p5, NGS primer for sequencing cDNA-TR RNA hybrid for A mutagenesis assay |
| oNEB-461 | CTTTCCCTACACGACGCTCTTCCGATCTHMCTGCGTCCCTGTGGC | p5, NGS primer for sequencing cDNA-TR RNA hybrid for A mutagenesis assay |
| oNEB-462 | CTTTCCCTACACGACGCTCTTCCGATCTNCTGCGTCCCTGTGGC | p5, NGS primer for sequencing cDNA-TR RNA hybrid for A mutagenesis assay |
| oNEB-463 | CTTTCCCTACACGACGCTCTTCCGATCCTGCGTCCCTGTGGC | p5, NGS primer for sequencing cDNA-TR RNA hybrid for A mutagenesis assay |
| oNEB-528 | CTTTCCCTACACGACGCTCTTCCGATCTVHDBGCGCTGCGTCCCTG | p7, NGS primer for sequencing DGR reporter strength assay |
| oNEB-529 | CTTTCCCTACACGACGCTCTTCCGATCTHMGCGCTGCGTCCCTG | p7, NGS primer for sequencing DGR reporter strength assay |
| oNEB-530 | CTTTCCCTACACGACGCTCTTCCGATCTNGCGCTGCGTCCCTG | p7, NGS primer for sequencing DGR reporter strength assay |
| oNEB-531 | CTTTCCCTACACGACGCTCTTCCGATCTGCGCTGCGTCCCTG | p7, NGS primer for sequencing DGR reporter strength assay |
| oNEB-535 | CTTTCCCTACACGACGCTCTTCCGATCTVHDBATCGCCTTCTATCGCCTTCT | p5, NGS primer for sequencing cDNA-TR RNA hybrid |
| oNEB-536 | CTTTCCCTACACGACGCTCTTCCGATCTVHDBATCGCCTTCTATCGCCTTCT | p5, NGS primer for sequencing cDNA-TR RNA hybrid |
| oNEB-537 | CTTTCCCTACACGACGCTCTTCCGATCTNATCGCCTTCTATCGCCTTCT | p5, NGS primer for sequencing cDNA-TR RNA hybrid |
| oNEB-538 | CTTTCCCTACACGACGCTCTTCCGATCATCGCCTTCTATCGCCTTCT | p5, NGS primer for sequencing cDNA-TR RNA hybrid |
| Cloning Primers | | |
| oNEB-32 | CACATTCTTGCATTCGACGTGCTGCGCCTTTATTTATCACCAGCAGGAAACGCCTTGTCCGCCAGTGCCAAGCTTGCATG | pF for insertion of kan reporter at 156 degrees. Amplification from pNEB34 or pNEB35 |
| oNEB-33 | AGGCGCAATTGTGTCACTGGAATAGAGGTTTCTTGTGGCGCATGTGGAAGGGGCGTCTATATGGGAATTAGCCATGGTCC | pR for insertion of kan reporter at 156 degrees. Amplification from pNEB34 or pNEB35 |
| oNEB-192 | CTGTCTCTTATACACATCTGTAGGCTGGAGCTGCTTCG | pF for addition of Tn5 sites. Amplification from pNEB34 or pNEB35 |
| oNEB-193 | CTGTCTCTTATACACATCTGATGGGAATTAGCCATGGTCC | pR for addition of Tn5 sites. Amplification from pNEB34 or pNEB35 |
| oNEB-219 | TAAATGATCATTTCCACGACGACGCGTAAAGCCAATGCGGTCAAAATATTCCAGATGTAGGCTGGAGCTGCTTCG | pF for insertion of kan reporter at 291 degrees. Amplification from pNEB34 or pNEB35 |
| oNEB-220 | GCGCGGCGGATTTCCGCGATCGCTTAGGCGTAGGCCGAAAGCTGGCAATTCAGATATGGGAATTAGCCATGGTCC | pR for insertion of kan reporter at 291 degrees. Amplification from pNEB34 or pNEB35 |
| oNEB-221 | AACGTTTTTGATGATAAATGCAGTGTCGGATGCGGCGCATCCGACACAGTTCACCTGTAGGCTGGAGCTGCTTCG | pF for insertion of kan reporter at 317 degrees. Amplification from pNEB34 or pNEB35 |
| oNEB-222 | GAGATTATTGCCGGGTTAACAGGATATGAGGCATTTATGGAGTTACTTCACAAGGATGGGAATTAGCCATGGTCC | pR for insertion of kan reporter at 317 degrees. Amplification from pNEB34 or pNEB35 |
| oNEB-225 | TACGCTTATCAGGCCTACGTGAACTCTGCAATATATTGAATTTGCATGCTTTTGTTGTAGGCTGGAGCTGCTTCG | pF for insertion of kan reporter at 63 degrees. Amplification from pNEB34 or pNEB35 |
| oNEB-226 | CTGACAAGTGCTTGTTGTGAAATGCCGGATGCGGCGTAAACGCCTTATCCGGCCTATGGGAATTAGCCATGGTCC | pR for insertion of kan reporter at 63 degrees. Amplification from pNEB34 or pNEB35 |
| oNEB-857 | CGCCTGGAGCTCTGGACATTAAACCAGGAGCTGGCTGCTAAGTGATTTAACGGCTTAAGTGCCGAAGAGCGATCG | *dnaG(K580A)* recombineering |
| qPCR Primers | | |
| oNEB-300 | CGCATCGCCTTCTATCGC | pF to quantify TR RNA |
| oNEB-301 | CCTTCTTGCATGGCTCTGC | pF to quantify the cDNA-TR RNA hybrid |
| oNEB-313 | GGAAGGCGTAGAGATGGTAATG | pF to quantify tufA |
| oNEB-314 | GCCCAGAACTTTAGCAACAAC | pR to quantify tufA |
| oNEB-343 | CGCCAAGCCGTAGCGTTG | RT primer to quantify TR RNA |
| oNEB-344 | CGCATCGCCTTCTATC | RT primer to detect the cDNA-TR RNA hybrid |
| oNEB-345 | ATCGCCTTCTATCGCCTTCT | pR to quantify the cDNA-TR RNA hybrid |
| oNEB-346 | GCCGTAGCGTTGGCAGAG | pR to quantify TR RNA |
| oNEB-356 | GCGCTGCGTCCCTG | RT primer to sequence the cDNA-TR RNA hybrid for A mutagenesis experiments |
| oNEB-573 | CCCTGTGGCAAAGGATATCAA | pF to measure promoter strength of the reporter |
| oNEB-574 | GCTACTCAAGAATCAGGTGGTC | pR to measure promoter strength of the reporter |
|  |  |  |
