# Supplementary material for: High-throughput analyses of a reconstituted diversity-generating retroelement identify intrinsic and extrinsic determinants of diversification
Source: PLoS Genet. 2026 Feb 5;22(2):e1012038. doi: 10.1371/journal.pgen.1012038 (PMC12875486; doi:10.1371/journal.pgen.1012038)
Supplement: S1 File — (DOCX) [file pgen.1012038.s014.docx]

**SUPPORTING INFORMATION**

**High-throughput Analyses of a Reconstituted Diversity-Generating Retroelement Identify Intrinsic and Extrinsic Determinants of Diversification**

Irem Unlu^ψ^, Marina K. Smiley^ψ^, Vladimir Potapov, Yoan Renoux-Martin, Zhi-Yi Sun, Hoong Chuin Lim^*^

^ψ^These authors contributed equally to this work.

^*^Corresponding author. E-mail : hlim@neb.com

**Supplementary Materials and Methods**

*E. coli* strain construction

*dnaG point mutant:* The *E. coli* strain bearing the *dnaG(K580A)* point mutation was generated using the Millipore Sigma CRISPR-Recombineering system, following the manufacturer's protocol. Briefly, electrocompetent cells containing the Cas9-Recombineering plasmid (CAS9BAC1P) were transformed with a guide RNA (gRNA) plasmid targeting the *dnaG* gene and a single-stranded DNA (ssDNA) oligo containing the desired mutation. After recovery in SOC medium, the culture was plated on LB agar with Amp100 and incubated for 3 days at room temperature. Clones were screened for the mutation using PCR and Sanger sequencing. Positive strains were subsequently plasmid-cured by plating on LB agar plates supplemented with 10% sucrose and incubating at 37°C overnight.

Immunoblotting

Cell pellets (harvested from 2.5 ml of ~2 OD600 cultures) were mixed with 200 µl of NEB 1X Blue Protein Loading Dye containing 1X DTT (NEB, B7703), sonicated briefly and boiled at 100°C for 5 minutes. 10 µl of the lysates were run on Novex 4-20% Tris-Glycine gel (Invitrogen, XP04205BOX) at 130V for 1 hour and transferred with BioRad Trans-Blot Turbo Transfer System with Mixed MW setting. Membranes were incubated overnight with 1:1500 Alexa647 conjugated anti-ALFA single domain antibody (NanoTag, N1502-AF647-L), washed three times with PBST and imaged or incubated overnight with 1:100,000 Anti-*E. coli* RNA Polymerase β antibody (BioLegend, 662904), washed three times with PBST and incubated with HRP-linked Anti-mouse IgG (Cell Signaling, 7076S) as the secondary antibody, washed three times with PBST and imaged. Imaging was done with LICOR Odyssey M.
